# Persistent and Emerging High‐Risk Clusters of Leprosy Detection in Brazil: A Nationwide Spatiotemporal Analysis, 2001–2023

**DOI:** 10.1111/tmi.70104

**Published:** 2026-02-16

**Authors:** Anderson Fuentes Ferreira, Jorg Heukelbach, Eliana Amorim de Souza, Carmelita Ribeiro Filha Coriolano, Elaine Silva Nascimento Andrade, Aymée Medeiros da Rocha, José Alexandre Menezes da Silva, Carlos Dornels Freire de Souza, Juliana Maria Cavalcante Ribeiro Ramos, Alberto Novaes Ramos

**Affiliations:** ^1^ Postgraduate Program in Public Health, School of Medicine Federal University of Ceará Fortaleza Ceará Brazil; ^2^ Multidisciplinary Institute of Health, Federal University of Bahia – Anísio Teixeira Campus Vitória da Conquista Bahia Brazil; ^3^ Rondônia State Health Surveillance Agency Porto Velho Rondônia Brazil; ^4^ Oswaldo Cruz Foundation Eusébio Ceará Brazil; ^5^ NHR Brazil Foundation Fortaleza Ceará Brazil; ^6^ CUIDA Chagas Project Rio de Janeiro Rio de Janeiro Brazil; ^7^ Federal University of Vale Do São Francisco Petrolina Pernambuco Brazil; ^8^ Fortaleza Municipal Health Department Fortaleza Ceará Brazil; ^9^ Department of Community Health, School of Medicine Federal University of Ceará Fortaleza Ceará Brazil

**Keywords:** Brazil, disease clusters, ecological studies, epidemiology, leprosy, spatial distribution

## Abstract

**Background:**

Brazil accounts for a substantial share of the global leprosy burden, with persistent high‐risk areas and marked spatial inequalities in case detection. Identifying spatiotemporal clusters of transmission is crucial for targeting surveillance and control efforts.

**Objective:**

To identify the spatial and temporal variation of high‐risk clusters for leprosy detection in Brazil (2001–2023) and priority areas for intervention.

**Methods:**

This was a nationwide, population‐based ecological study using Brazilian municipalities (*n* = 5570) as units of analysis. Frequencies, crude detection rates and age‐ and sex‐adjusted detection rates of newly recorded leprosy cases were calculated, based on the national Notifiable Diseases Information System (SINAN). Kulldorff's spatial scan statistic (Poisson model) was applied to overlapping three‐year periods to identify statistically significant high‐risk clusters. Neighbouring categories and changes between consecutive triennia were compared to classify municipalities into clusters based on persistence, entry, or exit.

**Results:**

Over the study period, a total of 795,802 cases were reported (age‐ and sex‐adjusted detection rate: 17.58/100,000 population; 95% CI: 17.54–17.62), with higher occurrence among males and residents of the Northeast region. Significantly high‐risk clusters were concentrated in the North, Northeast, and Central‐West regions; approximately 23% of municipalities were part of significant clusters in each triennium. The proportion of municipalities newly entering clusters increased from 10.7% (2003–2005 vs. 2001–2003) to 36.0% (2021–2023 vs. 2019–2021). In the most recent triennium (2021–2023), 16.9% of municipalities remained within significant clusters.

**Conclusion:**

The persistence and (re)emergence of high‐risk leprosy clusters in historically hyperendemic and socially vulnerable territories indicate sustained operational failures and demand programmatic prioritisation, with intensified surveillance, timely diagnosis and adequate treatment, particularly in the North, Northeast and Central‐West regions of Brazil.

## 1 Introduction

Leprosy is strongly associated with social vulnerability, poverty, and stigma. It is classified among the Neglected Tropical Diseases (NTDs) [1] and shows a focal distribution with distinct levels of endemicity across territories [2]. Globally, reported new cases (NC) declined from 214,339 in 2014 to 172,717 in 2024, with a marked drop in 2020 (128,421 NC) during the SARS‐CoV‐2 pandemic. Reductions were observed in the two regions with the highest burden, South–East Asia (154,834 NC in 2014 to 124,295 in 2024) and the Region of the Americas (33,789 NC in 2014 to 23,593 in 2024), where Brazil accounted for over 90% of reported cases. In 2024, India (100,957; 58.5%), Brazil (22,129; 12.8%), and Indonesia (14,698; 8.5%) were the countries with the highest number of NC [3]. The global reduction of NC during the most critical years of the COVID‐19 pandemic reached −36.6% in 2020 and −30.6% in 2021, compared to 2019 [3]. In Brazil, time‐trend analyses similarly showed declines in detection rates in the general population and among individuals under 15 years, reflecting the impact on leprosy control [4, 5].

Despite these declines, the persistence of high‐burden territories underscores the need to strengthen care, surveillance, prevention, and control. Leprosy cases concentrate in areas with worse social indicators [2, 6, 7] and in specific pockets within endemic states [8, 9], while factors such as population migration contribute to maintaining endemic areas [10]. Identifying areas with persistent clusters of cases over time is therefore critical for targeting control efforts, while also avoiding neglect of under‐detected “silent” areas [2].

The Brazilian National Leprosy Control Programme (*Programa Nacional de Controle da Hanseníase*, PNCH) has historically used evidence from spatial cluster analyses to define priority areas for surveillance and control [6, 11].

Spatial analyses are a key tool for leprosy control, particularly after 2 years of substantial disruption to surveillance and control activities due to COVID‐19 [12]. Integrated approaches to understanding leprosy dynamics in endemic areas underscore the need for robust spatiotemporal analyses to support programme planning [13]. In this context, the objective of this study was to analyse the spatial and temporal variation in high‐risk leprosy clusters in Brazil from 2001 to 2023. The definition of priority areas will facilitate the elaboration of more effective control measures and policies in territories where disease clusters persist, emerge, or disappear.

## 2 Methods

### 2.1 Study Design

We conducted an ecological study with spatial and temporal components to identify areas with high leprosy detection rates in Brazil during 2001–2023. The units of analysis were the 5570 Brazilian municipalities.

To reduce annual variability and obtain more robust estimates, we used to overlap three‐year periods (2001–2003, 2003–2005, 2005–2007, 2007–2009, 2009–2011, 2011–2013, 2013–2015, 2015–2017, 2017–2019, 2019–2021, 2021–2023). The triennia 2005–2007 and 2007–2009 had previously been used by the Ministry of Health as a reference for defining priority areas [6, 11].

### 2.2 Study Setting

**FIGURE 1:**
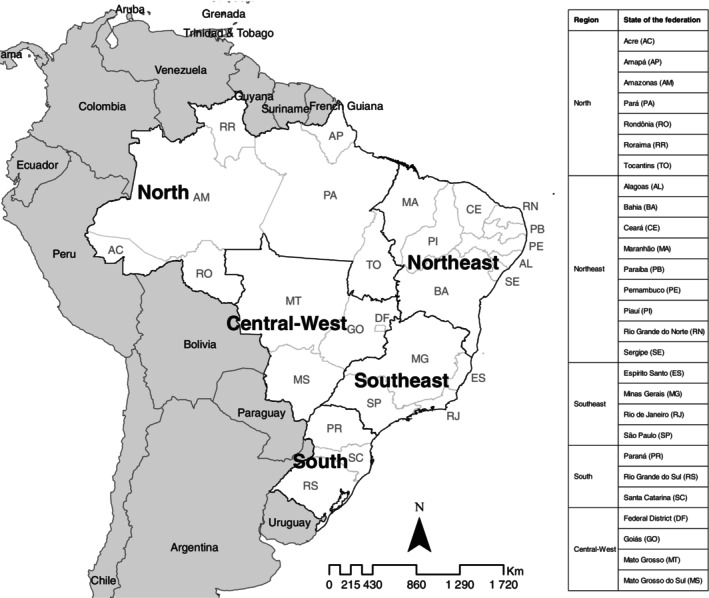
Study area: Brazilian states and regions.

Brazil is composed of 27 federal units (26 states and 1 Federal District) distributed across five major geographic regions (North, Northeast, Southeast, South and Central‐West), comprising 5570 municipalities (Figure 1). With a total area of 8,510,418 km^2^, the country had a population of 203,062,512 inhabitants in 2022 and a population density of 23.86 inhabitants/km^2^ [14].

Between 2000 and 2021, the Human Development Index (HDI) increased from 0.612 to 0.766, and life expectancy at birth rose from 68.61 to 74.16 years. Over the same period, the Gini index decreased from 0.640 to 0.544, indicating reductions of income inequality [15]. This context of marked socioeconomic inequalities and regional heterogeneity is central to understanding the distribution of leprosy in Brazil.

### 2.3 Inclusion Criteria, Variables and Data Sources

We included all new leprosy cases recorded in the Brazilian Notifiable Diseases Information System (*Sistema de Informação de Agravos de Notificação*, SINAN) among residents in Brazil, with a date of diagnosis between January 2001 and December 2023. Records with ‘diagnostic error’ as an exit criterion and those with unknown municipality of residence were excluded.

We first calculated absolute and relative frequencies of demographic and clinical variables for new cases, including: sex (male, female); age group in years (< 15, 15–29, 30–39, 40–49, 50–59, 60–69, ≥ 70); ethno‐racial group (Caucasian, Afro‐Brazilian, Asian, Mixed/Pardo‐Brazilian, Indigenous [Amerindians]—based on self‐reported race/skin colour according to the Brazilian census classification); region of residence (North, Northeast, Southeast, South, Central‐West); rural–urban typology (urban, intermediate adjacent, intermediate remote, rural adjacent, rural remote); municipality type, according to a classification aligned with the National Health Survey (*Pesquisa Nacional de Saúde*, PNS) [16]: large and aggregated [Metropolitan Region (*Região Metropolitana*, RM), Integrated Development Region (*Região Integrada de Desenvolvimento*, RIDE) or urban clusters (AGLO)], medium and aggregated (RM/RIDE/AGLO), small and aggregated (RM/RIDE/AGLO), large and isolated, medium and isolated, small and isolated; Brazilian Deprivation Index (*Índice Brasileiro de Privação*, IBP) [17] (reference year 2010: very low, low, medium, high, very high); epidemiological and operational groups [18]; operational classification (paucibacillary [PB], multibacillary [MB]); grade of physical disability (GPD) at diagnosis (GPD0, GPD1, GPD2); mode of detection (referral, self‐presentation, group examination, contact examination, other). All missing values were identified and described with their respective percentages.

Rural–urban typology was defined using population size and degree of urbanisation [19]:

- Predominantly urban municipalities: 50,000 inhabitants in densely occupied areas; 25,000–50,000 inhabitants in densely occupied areas and urbanisation > 50%; 10,000–25,000 inhabitants in densely occupied areas and urbanisation > 75%.
- Intermediate municipalities: 25,000–50,000 inhabitants in densely occupied areas and urbanisation 25%–50%; 10,000–25,000 inhabitants in densely occupied areas and urbanisation 50%–75%; 3000–10,000 inhabitants in densely occupied areas and urbanisation 50%–75%.
- Predominantly rural municipalities: 25,000–50,000 inhabitants in densely occupied areas and urbanisation < 25%; 10,000–25,000 inhabitants in densely occupied areas and urbanisation < 50%; 3000–10,000 inhabitants in densely occupied areas and urbanisation < 75% [19].

Municipality size was defined as [20]: ‘small’: population < 50,000 inhabitants and population density < 80 inhabitants/km^2^; “medium”: population 50,000–100,000 inhabitants and population density ≥ 80 inhabitants/km^2^; and “large”: population > 100,000 inhabitants, regardless of population density.

The Brazilian Deprivation Index (IBP) was used as a synthetic measure of material deprivation and socioeconomic position at the municipal level. The index is based on three dimensions [17, 21]: income (percentage of households with per capita income < ½ minimum wage); education (percentage of illiterate individuals aged ≥ 7 years); housing conditions (percentage of households with inadequate access to basic sanitation and without piped water, waste collection, toilet and bathroom), using 2010 census data.

The 2019–2022 classification of epidemiological and operational groups was based on new cases notified from 2013 to 2017, with the aim of identifying priority municipalities for leprosy prevention and control [18]. Municipalities were grouped as: Group 1: no cases notified during the period; Group 2: new case detection rate < 10/100,000 inhabitants; Group 3: new case detection rate ≥ 10/100,000 inhabitants.

Groups 2 and 3 were further stratified into four subgroups: < 75% of cases assessed for GPD at diagnosis; ≥ 75% of cases assessed for GPD at diagnosis, presence of cases with GPD2, and < 80% of contacts examined; ≥ 75% of cases assessed for GPD at diagnosis, presence of cases with GPD2, and ≥ 80% of contacts examined; ≥ 75% of cases assessed for GPD at diagnosis and no cases with GPD2 [18].

In the “National Strategy for Confronting Leprosy 2024–2030,” municipalities were reclassified into two main groups [22]: Group 1: without leprosy cases; Group 2: with leprosy cases.

Group 2 was subdivided into three categories: 2.1: municipalities with sporadic cases; 2.2: municipalities with cases only in individuals aged ≥ 15 years; 2.3: municipalities with cases in individuals aged < 15 years.

Groups 2.2 and 2.3 were then stratified according to operational indicators [22]:

- Group 2.2 (no cases in < 15 years)—2.2.1: ≥ 75% of cases assessed for GPD and low percentage of GPD2; 2.2.2: < 75% of cases assessed for GPD; and 2.2.3: ≥ 75% of cases assessed for GPD and high percentage of GPD2.
- Group 2.3 (with cases in < 15 years)—2.3.1: ≥ 75% of cases assessed for GPD and low percentage of GPD2; 2.3.2: < 75% of cases assessed for GPD; and 2.3.3: ≥ 75% of cases assessed for GPD and high percentage of GPD2 [22].

In addition to crude detection rates, we calculated age‐ and sex‐adjusted detection rates using the direct standardisation method, with 95% confidence intervals (95% CI).

The SINAN dataset was formally obtained from the General Coordination of Leprosy and Diseases in Elimination (*Coordenação‐Geral de Hanseníase e Doenças em Eliminação*, CGHDE) of the Department of Surveillance of Communicable Diseases, Secretariat of Health Surveillance, Ministry of Health of Brazil (CGHDE/DEVIT/SVS/MS).

Population data were obtained from the Brazilian Institute of Geography and Statistics (*Instituto Brasileiro de Geografia e Estatística*, IBGE), using the 2000, 2010, and 2022 census data and intercensal population estimates (2001–2009, 2011–2021, 2023). Digital cartographic files used for thematic mapping and spatial analyses were also obtained from IBGE, based on the 2022 territorial division.

### 2.4 Spatial Analyses

For spatial analysis, municipalities of residence (*n* = 5570, territorial division of 2022) were used as units of analysis. Kulldorff's spatial scan statistic was applied to identify high‐risk clusters in each triennium [23]. Given that leprosy is a relatively rare event in the general population, we used a discrete Poisson probability model.

The following parameters were specified: no geographical overlap between clusters, circular scanning window, maximum radius of 500 km, and maximum cluster size of 50% of the population at risk. Primary and secondary clusters were identified using the log‐likelihood ratio (LLR), and statistical significance was assessed with 99,999 Monte Carlo simulations. For this study, statistically significant clusters were defined as those including at least three contiguous municipalities.

The definition of cluster and neighbourhood categories followed four steps:

1. generation of clusters for each triennium using Kulldorff's scan statistic;
2. identification of municipalities neighbouring clusters, based on spatial contiguity;
3. classification of municipalities according to their position in clusters (significant or non‐significant) or in cluster neighbourhoods (significant or non‐significant), as well as those not included in clusters or neighbourhoods;
4. categorisation of municipalities into five situations:
  ○ not included in a cluster and not neighbouring any cluster;
  ○ neighbour of a non‐significant cluster;
  ○ included in a non‐significant cluster;
  ○ neighbor of a significant cluster;
  ○ included in a significant cluster.

Municipality‐level thematic maps were produced to display clusters and their neighbourhoods for each triennium, as well as comparative maps between periods to assess temporal changes.

Descriptive statistical analyses were performed using Stata, version 11.2 (StataCorp LLC, College Station, TX, USA). SaTScan (Martin Kulldorff, Harvard Medical School, Boston; Information Management Services Inc., Silver Spring, MD, USA) was used to detect spatial clusters. QGIS 2.18.6 (https://qgis.org/pt_BR/site/) was used for thematic mapping.

### 2.5 Inter‐Triennium Comparison

To identify spatiotemporal changes, we compared consecutive triennia, using 2001–2003 as the baseline period. For each pair of consecutive triennia, municipalities were classified as:

- never included in a cluster;
- neighbour of a cluster but not included;
- no longer included in a cluster (significant or non‐significant);
- newly included in a cluster (significant or non‐significant);
- remained in a significant cluster.

This classification allowed us to examine the persistence, entry, and exit of municipalities in high‐risk clusters over time.

### 2.6 Ethics Statement

This national study was based exclusively on secondary, aggregate, or anonymised data and did not involve direct contact with persons affected by leprosy or their families/communities, nor did it involve access to individually identifiable information from medical records or nominal notification forms. This study complied with ethical principles for research in Brazil, in accordance with National Health Council Resolution No. 466 of 12 December 2012 and the General Personal Data Protection Law (*Lei Geral de Proteção de Dados Pessoais*, LGPD), Law No. 13,709 of 14 August 2018.

All data were obtained in accordance with protocols established by the Ministry of Health of Brazil.

## 3 Results

Over the 23‐year study period, a total of 795,802 new leprosy cases were recorded in Brazil, with an age‐ and sex‐adjusted detection rate of 17.58/100,000 population (95% CI 17.54–17.62). Most cases occurred in males (55.1%; *N* = 438,341), individuals aged 15–29 years (21.0%; *N* = 167,488) and those self‐identified as Mixed/Pardo Brazilians (48.3%; *N* = 384,441). The highest detection rates were observed among males (adjusted rate 19.80/100,000; 95% CI 19.74–19.86), individuals aged 60–69 years (adjusted rate 32.31/100,000; 95% CI 32.11–32.52) and Afro‐Brazilians (crude rate 27.94/100,000) (Table 1).

**TABLE 1:** Absolute numbers and proportions of new leprosy cases, crude and age‐ and sex‐adjusted detection rates (per 100,000 inhabitants), by sociodemographic and clinical variables, Brazil, 2001–2023.

Variables	New cases	Crude rate	Age‐and sex–adjusted rate (95% CI)
	*N* (%)		
Total of cases	795,802 (100.0)	17.75	17.58 (17.54–17.62)
Sex			
Male	438,341 (55.1)	19.95	19.80 (19.74–19.86)
Female	357,307 (44.9)	15.63	15.45 (15.40–15.50)
Missing data	154 (0.0)	—	
Age group			
< 15	56,209 (7.1)	5.08	5.17 (5.13–5.22)
15–29	167,488 (21)	14.00	14.39 (14.32–14.46)
30–39	134,638 (16.9)	19.49	19.09 (18.98–19.19)
40–49	144,036 (18.1)	24.87	24.38 (24.25–24.50)
50–59	134,254 (16.9)	31.27	29.83 (29.67–29.99)
60–69	94,902 (11.9)	35.87	32.31 (32.11–32.52)
≥ 70	64,271 (8.1)	29.84	27.10 (26.89–27.31)
Missing data	4 (0.0)	—	—
Ethnoracial group			
Caucasian	212,354 (26.7)	10.14	—
Afro‐Brazilian/Afro‐descendant	93,297 (11.7)	27.94	—
Asian‐descendant	9592 (1.2)	20.01	—
Mixed/Pardo Brazilians	384,441 (48.3)	20.32	—
Indigenous (Amerindians)	2846 (0.4)	15.13	—
Missing data	93,272 (11.7)	—	—
Region of residence			
North	158,343 (19.9)	42.38	46.23 (46.00–46.47)
Northeast	322,115 (40.5)	25.83	26.92 (26.83–27.01)
Southeast	143,681 (18.1)	7.60	7.23 (7.19–7.27)
South	30,760 (3.9)	4.79	4.43 (4.38–4.48)
Central–West	140,673 (17.7)	42.62	42.28 (42.06–42.51)
Missing data	230 (0.0)	—	—
Typology of municipality			
Urban	561,455 (70.6)	16.50	16.18 (16.14–16.23)
Intermediate adjacent	68,910 (8.7)	23.54	23.79 (23.61–23.96)
Intermediate remote	19,595 (2.5)	63.73	72.59 (71.55–73.64)
Rural adjacent	110,330 (13.9)	16.37	16.68 (16.58–16.78)
Rural remote	35,088 (4.4)	42.34	48.59 (48.07–49.10)
Missing data	424 (0.1)	—	—
Type of municipality (compatible for PNS)			
Large and aggregated (MR, IDRS or AGGL)	215,931 (27.1)	13.62	13.32 (13.27–13.38)
Medium and aggregated (MR, IDRS or AGGL)	14,617 (1.8)	12.61	12.66 (12.46–12.87)
Small and aggregated (MR, IDRS or AGGL)	15,326 (1.9)	24.39	24.75 (24.36–25.15)
Large and isolated	169,733 (21.3)	17.74	17.33 (17.24–17.41)
Medium and isolated	125,193 (15.7)	19.68	19.80 (19.69–19.91)
Small and isolated	254,608 (32)	22.63	22.95 (22.86–23.04)
Missing data	394 (0.0)	—	—
Brazilian index of deprivation			
Very low	40,868 (5.1)	4.97	4.56 (4.52–4.61)
Low	54,321 (6.8)	6.60	6.33 (6.28–6.38)
Medium	136,533 (17.2)	15.15	14.85 (14.77–14.93)
High	274,120 (34.4)	30.60	30.66 (30.55–30.78)
Very high	289,536 (36.4)	27.84	29.88 (29.77–29.99)
Missing data	424 (0.1)	—	—
Classification of epidemiological and operational groups—Strategy 2019–2022			
No cases	4022 (0.5)	2.35	2.19 (2.12–2.26)
Subgroup 2.1	1837 (0.2)	1.99	1.88 (1.79–1.97)
Subgroup 2.2	4574 (0.6)	2.32	2.22 (2.15–2.28)
Subgroup 2.3	13,438 (1.7)	2.19	2.10 (2.06–2.13)
Subgroup 2.4	2099 (0.3)	2.30	2.24 (2.14–2.33)
Subgroup 3.1	80,483 (10.1)	23.29	23.81 (23.65–23.98)
Subgroup 3.2	66,350 (8.3)	14.88	14.75 (14.63–14.86)
Subgroup 3.3	342,533 (43.0)	25.29	25.17 (25.08–25.25)
Subgroup 3.4	280,042 (35.2)	23.97	24.13 (24.04–24.22)
Missing data	424 (0.1)	—	—
Classification of epidemiological and operational groups—Strategy 2024–2030			
No cases	7183 (0.9)	2.80	2.62 (2.56–2.68)
Group 2.1	33,067 (4.2)	5.74	5.57 (5.51–5.63)
Group 2.2			
Subgroup 2.2.1	52,076 (6.5)	12.09	11.88 (11.78–11.98)
Subgroup 2.2.2	22,364 (2.8)	10.11	9.84 (9.71–9.97)
Subgroup 2.2.3	38,732 (4.9)	10.55	10.22 (10.12–10.32)
Group 2.3			
Subgroup 2.3.1	153,070 (19.2)	34.63	35.48 (35.30–35.66)
Subgroup 2.3.2	117,565 (14.8)	28.61	28.83 (28.66–28.99)
Subgroup 2.3.3	371,351 (46.7)	20.93	20.83 (20.76–20.90)
Missing data			
Operational classification of the disease			
Paucibacillary	303,739 (38.2)	—	—
Multibacillary	490,893 (61.7)	—	—
Missing data	1170 (0.1)	—	—
Degree of disability			
Grade zero	489,894 (61.6)	—	—
Grade I	165,373 (20.8)	—	—
Grade II	52,877 (6.6)	—	—
Not assessed	69,156 (8.7)	—	—
Missing data	18,502 (2.3)	—	—
Mode of detection of new cases			
Referral	341,375 (42.9)	—	—
Spontaneous demand	347,607 (43.7)	—	—
Community screening	26,043 (3.3)	—	—
Contact screening	61,069 (7.7)	—	—
Other modes	12,257 (1.5)	—	—
Missing data	7451 (0.9)	—	—

Abbreviations: %, percentage; —, not calculated; AGLO, urban agglomerations; CI, confidence interval; IBP, Brazilian Index of Deprivation (*Índice Brasileiro de Privação*); MR, Metropolitan Region; *N*, number; PNS, National Health Survey (Pesquisa Nacional de Saúde); RIDE: Integrated Development Region (*Região Integrada de Desenvolvimento*).

All Brazilian macro‐regions showed a reduction in detection rates after 2003, followed by a sustained increase from 2016 onwards and a sharp decline during the COVID‐19 pandemic years, when the lowest rates in the time series were observed. Rates subsequently increased again, particularly in the Central–West region. Throughout most of the period, the North, Central‐West and Northeast regions maintained detection rates above the national average (Figure S1). The Northeast concentrated the largest share of new cases (40.5%; *N* = 322,115), whereas the highest detection rates were observed in the North (adjusted rate 46.23/100,000; 95% CI 46.00–46.47), followed by the Central‐West (adjusted rate 42.28/100,000; 95% CI 42.06–42.51) (Table 1).

Municipalities classified as “Urban” accounted for the majority of new cases (70.6%; *N* = 561,455). However, the highest adjusted detection rates were observed in “Intermediate remote” municipalities (72.59/100,000; 95% CI 71.55–73.64) and “Rural remote” municipalities (48.59/100,000; 95% CI 48.07–49.10). According to the National Health Survey (*Pesquisa Nacional de Saúde*—PNS) municipal cluster classification, “Small and isolated” (32.0%; *N* = 254,608) and “Large and aggregated” municipalities (27.1%; *N* = 215,931) concentrated the majority of new cases, while the highest adjusted rate was found in “Small and aggregated” municipalities (24.75/100,000; 95% CI 24.36–25.15) (Table 1).

Municipalities with higher levels of material deprivation also showed a greater burden of disease. The highest proportions and adjusted detection rates of new cases were observed in areas classified as having “High” deprivation (34.4%; *N* = 274,120; adjusted rate 30.66/100,000; 95% CI 30.55–30.78) and “Very high” deprivation (36.4%; *N* = 289,536; adjusted rate 29.88/100,000; 95% CI 29.77–29.99) (Table 1).

Under the epidemiological and operational classification of the 2019–2022 National Strategy, almost all new cases occurred in group 3 (96.7%; *N* = 769,408). Within this group, subgroups 3.3 (43.0%; *N* = 342,533; adjusted rate 25.17/100,000; 95% CI 25.08–25.25) and 3.4 (35.2%; *N* = 280,042; adjusted rate 24.13/100,000; 95% CI 24.04–24.22) showed the largest concentrations (Table 1). In the 2024–2030 Strategy, group 2.3 concentrated most new cases (80.7%; *N* = 641,986), particularly subgroups 2.3.3 (46.7%; *N* = 371,351; adjusted rate 20.83/100,000; 95% CI 20.76–20.90) and 2.3.1 (19.2%; *N* = 153,070; adjusted rate 35.48/100,000; 95% CI 35.30–35.66) (Table 1).

Multibacillary forms accounted for 61.7% of new cases (*N* = 490,893), 6.6% (*N* = 52,877) presented Grade 2 disability at diagnosis, but 8.7% (*N* = 69,156) had no recorded disability assessment. Only 7.7% of new cases (*N* = 61,069) were detected through contact examination (Table 1).

**FIGURE 2:**
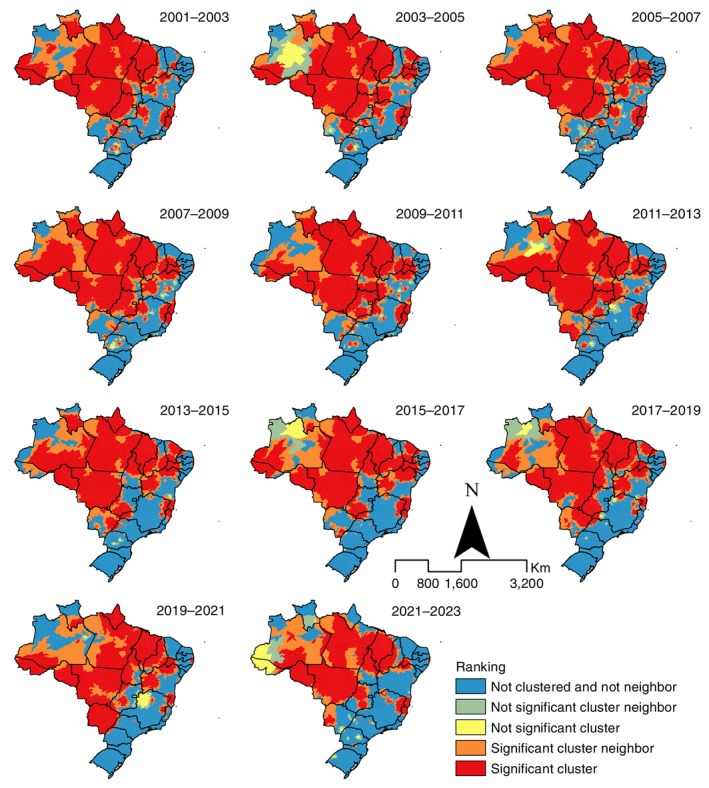
Clusters of new leprosy case detection rates, Brazil, 2001–2023.

Most high‐risk clusters, whether statistically significant or not, were identified in states in the North, Northeast and Central‐West regions. The South (mean 1.1% of municipalities across periods) and Southeast (mean 9.1%) regions consistently presented few municipalities within significant clusters throughout the study period (Figure 2; Table S1).

**FIGURE 3:**
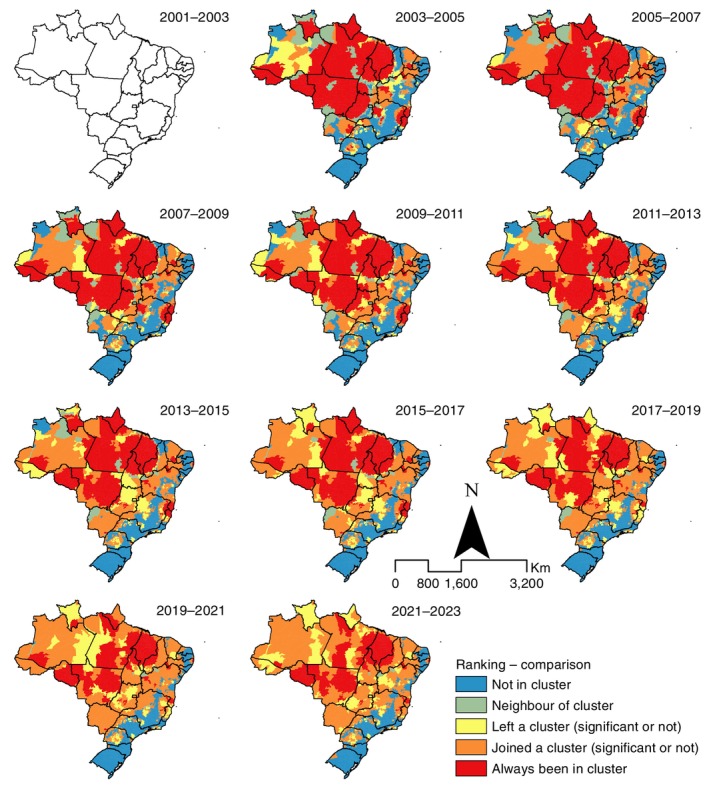
Temporal evolution and comparative patterns of clusters of new leprosy case detection rates, Brazil, 2001–2023. The 2001–2003 map is left blank because this period was used as the baseline for inter‐triennium comparisons. Changes in cluster patterns are shown from 2003 to 2005 onwards relative to 2001–2003.

**FIGURE 4:**
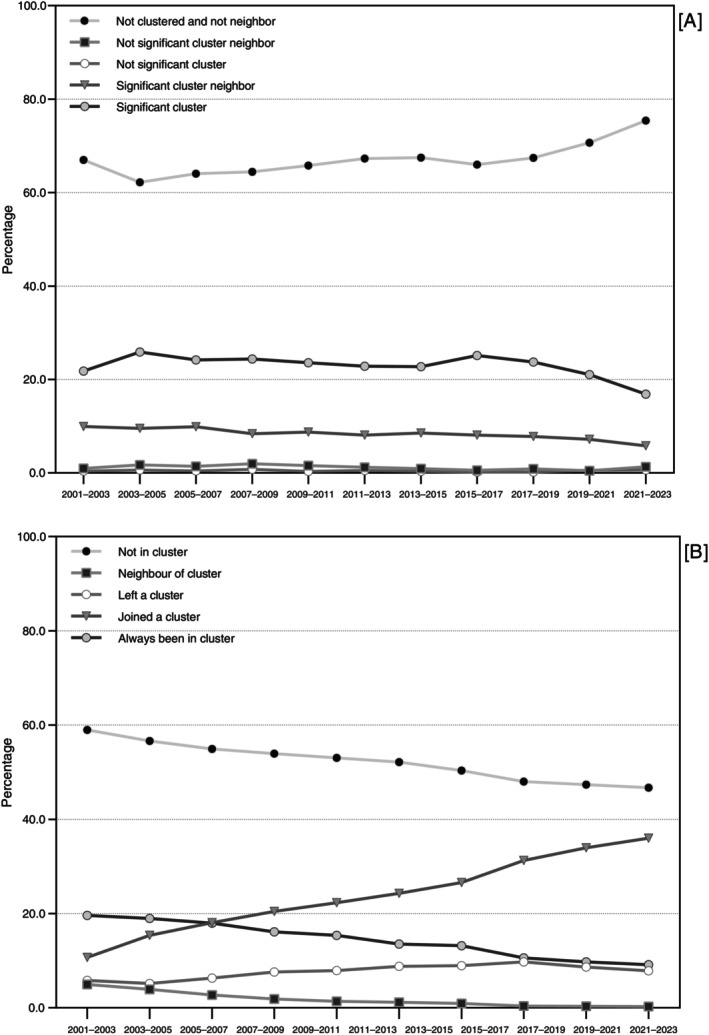
Temporal classification of municipalities: (A) spatial cluster analysis; (B) comparative evolution of clusters, Brazil, 2001–2023.

The analysis of cluster evolution over time showed a gradual reduction in the extent of areas with statistically significant clusters, but with persistent clusters in municipalities of the North and Northeast, especially in the states of Amapá, Pará, Acre (up to 2013), Rondônia, central Mato Grosso, Maranhão and western Piauí. Additional municipalities newly entering clusters were identified in Amazonas, Ceará, Mato Grosso do Sul, northern Bahia, northern Minas Gerais and southern Piauí. Most of these municipalities belonged to epidemiological and operational group 3 under the 2019–2022 Strategy and to group 2.3 under the 2024–2030 Strategy (Figures 3 and 4; Tables [Link], [Link]).

Throughout almost the entire study period, more than 20% of Brazilian municipalities were part of statistically significant clusters, with the exception of 2021–2023 (16.9%). In contrast, non‐significant clusters represented less than 1% of municipalities. The proportion of municipalities neither belonging to clusters nor adjacent to them increased from 62.2% in 2003–2005 to 75.4% in 2021–2023 (Figure 4A). The North and Northeast regions had the highest proportions of municipalities in significant clusters, especially those classified in groups 3 (2019–2022 Strategy) and 2.3 (2024–2030 Strategy) (Tables [Link], [Link]).

When comparing consecutive triennia, we observed a progressive reduction in the proportion of municipalities classified as “non‐cluster” in both periods, from 59.0% in 2003–2005 to 46.7% in 2021–2023. A similar reduction occurred among municipalities that remained in clusters across both triennia, decreasing from 19.6% to 9.2% over the same period. Conversely, there was a marked increase in the proportion of municipalities that newly entered clusters between consecutive triennia, from 10.7% in 2003–2005 to 36.0% in 2021–2023 (Figure 4B; Tables S2 and S3).

## 4 Discussion

Leprosy case detection remained highly concentrated in high‐risk spatial clusters in Brazil for more than two decades, particularly in the North, Northeast, and Central‐West regions, territories marked by deep social inequalities. The persistence and recent expansion of these clusters indicate ongoing transmission and persistent operational weaknesses in surveillance, diagnosis and care. The predominance of multibacillary cases, relevant levels of disability at diagnosis, incomplete disability assessment, and the limited role of contact examination in case detection further suggest delayed diagnosis and a substantial hidden burden of disease in vulnerable municipalities. Our nationwide analysis confirmed the persistence of classic high‐risk territories and revealed the entry of new municipalities into high‐risk clusters over time. These findings indicate that, despite important control efforts, transmission remains entrenched in socially vulnerable settings and has expanded to additional areas, posing major challenges for leprosy elimination in Brazil.

In 2023, the Central‐West (35.21/100,000 inhabitants) and North (18.78/100,000 inhabitants) regions had the highest general detection rates, with the same pattern observed in individuals under 15 years (Central‐West: 6.53/100,000; North: 4.09/100,000) and in cases with grade 2 disability (G2D) (Central‐West: 36.51/1,000,000; North: 17.77/1,000,000) [24].

Spatial analysis methods can deepen understanding of disease distribution at local and regional levels and allow risk estimation in areas without reported cases but with expected occurrence [25]. Such analyses can identify areas of greater severity and support strategic planning of context‐sensitive public policies [26], provided that studies are conducted with high methodological quality to inform decisions on surveillance and control [27]. The choice of parameters in spatial methods directly affects the selection of priority areas characterized by high case concentrations and/or elevated detection rates [28, 29].

A higher proportion of new cases was observed among men, consistent with previous national and state‐level studies [9, 30]. Beyond higher detection, male gender has been associated with lower rates of contact examination [30] and a greater risk of leprosy‐related mortality [31, 32]. This pattern highlights intersecting individual, social, and programmatic vulnerabilities that constrain timely access to health services and increase the probability of late diagnosis and worse outcomes [33].

New cases also occurred predominantly among adults aged 15–60 years [9, 34]. While the occurrence of leprosy in children and adolescents is a recognised marker of recent and active transmission, disease in older and economically active age groups signals accumulated exposure and heightened risk of physical disability and death from complications [31, 35]. These age patterns underscore the dual burden of ongoing transmission and long‐term sequelae, with implications for labour capacity, income and social protection.

The majority of new cases occurred among individuals self‐identified as Mixed/Pardo‐Brazilian, and the highest detection rates were observed among those self‐identified as Afro‐Brazilian [9, 34]. Ethno‐racial categories in Brazil are powerful markers of social stratification and structural inequities [36], and the concentration of leprosy among Afro‐Brazilian, Mixed/Pardo‐Brazilian, and Indigenous populations mirrors broader patterns observed for other NTDs [37]. These results reinforce that leprosy remains deeply embedded in the social fabric of inequality and racism, demanding responses that go beyond biomedical approaches and explicitly address social and racial justice.

At regional level, the macro‐areas with the highest proportions and rates of new cases correspond to those with worse sociodemographic indicators and higher levels of social vulnerability [37], reinforcing the link between poverty, precarious living conditions and disease occurrence [38]. Consistently, higher proportions of new cases were found in municipalities with greater material deprivation, as measured by the Brazilian Deprivation Index (IBP), emphasizing the central role of social determinants in sustaining leprosy transmission [9, 38]. Conversely, better socioeconomic conditions tend to align with improved health status and stronger performance of health systems [39].

Most new cases were concentrated in municipalities classified as non‐aggregated (outside metropolitan regions, integrated development regions or large urban clusters), as reported in previous studies [9]. In the context of a disease closely associated with social vulnerability, unplanned urban expansion and lack of regional integration amplify critical situations of poverty, unemployment, substandard housing, household overcrowding, intense human mobility and environmental degradation [40]. These conditions contribute to maintaining transmission and reinforce the need for territorial approaches to leprosy control.

We also observed a higher burden of disease in municipalities with larger populations and predominantly urban profiles, in line with earlier analyses [38]. However, new cases in rural areas deserve particular attention, given evidence of lower proportions of contact examination in these settings [30]. Rural contexts involve specific combinations of operational, individual, and social vulnerability that must be incorporated into the planning of prevention and control actions [41].

Multibacillary forms predominated among new cases, as documented in other regions and studies in Brazil [31, 39, 40]. This pattern points to late diagnosis, longer periods of infectiousness and increased risk of disability and complications [42]. The distribution of disability grade at diagnosis corroborates this scenario and mirrors findings from previous investigations [43, 44]. At the same time, the substantial proportion of new cases without recorded disability assessment reveals operational weaknesses in the health‐care network, especially within primary health care (PHC), undermining a strategic pillar for early diagnosis, prevention and rehabilitation of disabilities [44, 45]. This calls for urgent strengthening of comprehensive care, with a central role for multiprofessional teams in primary care, including multidisciplinary teams (eMulti teams) [43, 44].

From a surveillance perspective, contact examination, group examinations, surveys and active case‐finding campaigns are key strategies [45]. Nevertheless, in practice, most new cases continue to be detected through passive mechanisms—referral or spontaneous demand—with only a modest contribution from contact examination, despite some recent progress. The proportion of cases detected via contact examination increased from 7.3% in 2014 to 11.7% in 2023, partly due to the incorporation of rapid tests [24, 45], but remains insufficient, as also shown in other national analyses [30]. Historical barriers such as stigma, fear, prejudice, and discrimination contribute to this situation, limiting the access of people affected and their contacts to PHC and favouring delayed diagnosis [46, 47].

The heterogeneous distribution of leprosy across Brazil, with concentrations in municipalities with worse sociodemographic conditions [38] and high endemicity parameters [11, 45], reinforces its neglected status and its strong association with poverty [9]. The persistence of high detection rates and spatial clusters over time encompasses both urban and rural profiles [48], underscoring the need for context‐specific strategies.

Municipalities that remain within clusters over several triennia reflect sustained transmission driven by operational failures and social determinants [9]. In contrast, reductions in rates and case numbers may indicate effective surveillance and control, but can also reflect underdiagnosis or underreporting [6]. Interpreting these trends, therefore, requires careful consideration of both programmatic advances and potential gaps in case detection.

Within this context, national strategies such as the “National Strategy for Confronting Leprosy 2019–2022” [18] and the “National Strategy for Confronting Leprosy 2024–2030” [22] represent important steps towards risk‐based prioritisation. However, full alignment with international guidelines for surveillance and control is still needed, including the systematic incorporation of recommended interventions such as chemoprophylaxis for contacts and the global leprosy *roadmap* [49, 50]. More intensive and integrated use of epidemiological and operational indicators [51] can strengthen surveillance and control, focusing on municipalities that play a central role in sustaining transmission [6, 7, 18, 22].

Comparing patterns across different time periods proved useful for identifying both classical high‐risk areas and new municipalities that joined clusters, providing relevant information for decision‐making [27]. Changes in cluster configuration may reflect improvements in living conditions in areas that move out of clusters, better surveillance quality, or population changes such as migration and urbanisation [2, 10, 29]. Municipalities that persistently remain in clusters warrant particular priority, as they require intensification, reorientation, and the continuity of prevention and control actions, given their high and sustained risk [52].

While areas with high concentrations of new cases are usually the main focus of interventions, non‐significant clusters, cluster‐neighbouring municipalities, and those that have ceased to be clusters also require attention and tailored strategies. These territories may reveal fragile control activities, underreporting, or underdiagnosis. Estimates suggest that around 10% of leprosy cases may not be registered [53], contributing to the hidden burden of disease and maintaining transmission across multiple regions of Brazil.

In addition, environmental and zoonotic exposures may contribute to maintaining endemicity in some settings; for example, wildlife hunting and consumption, particularly of armadillos, have been associated with increased risk of leprosy transmission in the tropical Americas, especially in contexts of habitat change, deforestation, and conversion of native vegetation into pastures [54].

Structuring more context‐sensitive actions, in line with national guidelines, can effectively reduce disease burden [45]. Spatial analysis methods are particularly useful for delineating risk areas [28], identifying foci of transmission and guiding targeted interventions [13], and have the potential to promote earlier detection and reduce disability [29]. In addition, deaths associated with leprosy, captured in mortality information systems, can be considered sentinel events that complement routine surveillance and point to failures in timely diagnosis and care [32].

Even though detection rates declined during the COVID‐19 pandemic years, the proportion of municipalities classified as “non‐cluster” continued to decrease, and additional municipalities entered high‐risk clusters. This pattern is worrisome for control strategies, given the chronic nature of leprosy [4] and the possibility that national and international guidelines for surveillance, care and elimination have not been fully implemented or sustained [45, 49, 50].

Our study is subject to limitations. Firstly, SINAN secondary surveillance data may be subject to incomplete reporting, misclassification and underreporting. Incompleteness or inconsistent recording of variables, particularly disability grade and mode of detection, may have affected the accuracy of some indicators. Although previous evaluations suggest that SINAN coverage for leprosy has been generally adequate and relatively stable across regions during the study period [55, 56], other studies have documented persistent weaknesses in completeness and data quality [57]. Differences in diagnostic capacity and reporting practices between regions, municipalities and over time may have influenced both detection rates and the spatial configuration of clusters. Secondly, the ecological design and the use of municipality‐level aggregates limit causal interpretation at the individual level and may have masked intra‐municipal heterogeneity. The spatial scan statistic employs circular windows, which may not perfectly capture irregularly shaped or elongated clusters, and results are sensitive to parameter choices (e.g., maximum cluster size and radius). Furthermore, the measures obtained are relative (e.g., relative risk within clusters) rather than absolute, and should be interpreted as tools for programmatic prioritisation rather than precise estimates of individual‐level risk. The modifiable areal unit problem and the use of municipal boundaries may also influence observed patterns, particularly in municipalities with large territories or low population density. Thirdly, population denominators were derived from census data and intercensal estimates, which may introduce uncertainty in some strata, especially in rapidly changing or hard‐to‐reach areas. The COVID‐19 pandemic likely affected both health service utilisation and surveillance activities, potentially distorting temporal trends in detection and the apparent evolution of clusters. These factors should be borne in mind when interpreting the reduction in detection rates during the pandemic period and the subsequent increase.

Despite these limitations, the integrated analysis of official Brazilian data over a 23‐year period, encompassing areas of both high and low endemicity, provides an important and relatively rare national overview of the spatiotemporal dynamics of leprosy. By identifying persistent and emerging clusters at the municipal level and relating them to social vulnerability and operational indicators, this study provides evidence to support more targeted and equitable policies for surveillance, prevention, and control, aligned with the goal of eliminating leprosy as a public health problem in Brazil.

## 5 Conclusion

Our findings underscore the need for explicit territorial prioritisation, grounded in primary health care, and for the systematic use of spatial analyses and operational indicators to guide a more responsive and equity‐oriented management of leprosy control. The Brazilian Ministry of Health and state and municipal health secretariats should implement differentiated responses according to risk profile, including intensified early detection (through contact examination, community surveys and field outreach) in municipalities that remain in, or newly enter, high‐risk clusters; strengthening of clinical capacity and routine assessment of disability grade at diagnosis; continuous active surveillance in “neighbouring areas” and in municipalities that have left clusters, in order to avoid underreporting and resurgence; and integration of spatial analysis dashboards into routine management, with sustained analytical cycles to enable timely reallocation of resources. The adoption and scaling‐up of additional evidence‐based strategies, such as chemoprophylaxis for contacts in line with international recommendations, should also be considered.

Anchored in the current national strategies for confronting NTDs, these measures are essential to reduce the burden of leprosy, prevent disability and mitigate social and territorial inequalities in endemic areas. Prioritising persistent and emerging clusters, without neglecting adjacent or formerly high‐risk municipalities, will be crucial to move Brazil closer to the goal of eliminating leprosy as a public health problem.

## Conflicts of Interest

The authors declare no conflicts of interest.

## Supporting information


**Figure S1:** Age‐ and sex‐adjusted detection rates (per 100,000 inhabitants) by region and for Brazil, 2001–2023.

**Table S1:** Temporal classification by regions of Brazil: spatial analysis of clusters and comparative evolution of clusters, 2001–2023.

**Table S2:** Classification of epidemiological and operational groups in the 2019–2022 Strategy in relation to clusters and their temporal evolution, Brazil, 2001–2023.

**Table S3:** Classification of epidemiological and operational groups in the 2024–2030 Strategy in relation to clusters and their temporal evolution, Brazil, 2001–2023.

## Data Availability

The data that support the findings of this study are available in DATASUS at https://datasus.saude.gov.br/transferencia‐de‐arquivos/. These data were derived from the following resources available in the public domain: SINAN, https://ftp.datasus.gov.br/dissemin/publicos/SINAN/DADOS.
