# Supplementary material for: Persistent and Emerging High‐Risk Clusters of Leprosy Detection in Brazil: A Nationwide Spatiotemporal Analysis, 2001–2023
Source: Trop Med Int Health. 2026 Feb 16;31(4):532–46. doi: 10.1111/tmi.70104 (PMC13050618; doi:10.1111/tmi.70104)

**Supplements - Figure 1:** Age- and sex-adjusted detection rates (per 100,000 inhabitants) by region and for Brazil, 2001–2023


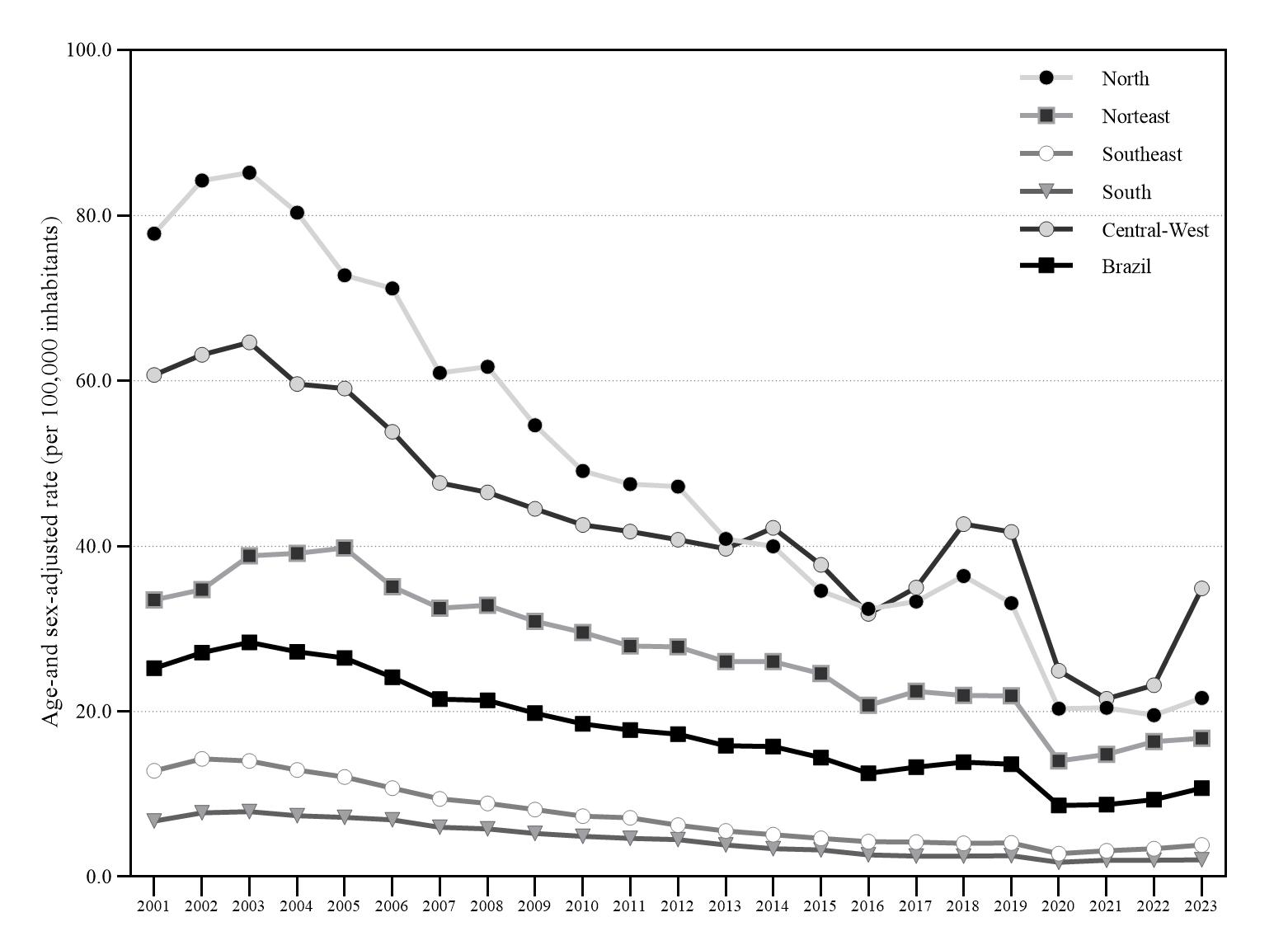

Supplement: Supplementary file 1 — Figure S1: Age‐ and sex‐adjusted detection rates (per 100,000 inhabitants) by region and for Brazil, 2001–2023. [file TMI-31-532-s002.docx]
