# Supplementary material for: Persistent and Emerging High‐Risk Clusters of Leprosy Detection in Brazil: A Nationwide Spatiotemporal Analysis, 2001–2023
Source: Trop Med Int Health. 2026 Feb 16;31(4):532–46. doi: 10.1111/tmi.70104 (PMC13050618; doi:10.1111/tmi.70104)
Supplement: Supplementary file 2 — Table S1: Temporal classification by regions of Brazil: spatial analysis of clusters and comparative evolution of clusters, 2001–2023. [file TMI-31-532-s001.docx]

**Supplements – Table 1:** Temporal classification by regions of Brazil: spatial analysis of clusters and comparative evolution of clusters, 2001–2023

| **Municipality classification** | **North** | | **Northeast** | | **Southeast** | | **South** | | **Central-West** | | **Brazil** | |
| --- | --- | --- | --- | --- | --- | --- | --- | --- | --- | --- | --- | --- |
|  | **N** | **%** | **N** | **%** | **N** | **%** | **N** | **%** | **N** | **%** | **N** | **%** |
| **Number of municipalities** | **450** | **8.1** | **1,794** | **32.2** | **1,668** | **29.9** | **1,191** | **21.4** | **467** | **8.4** | **5,570** | **100.0** |
| **Ranking** |  |  |  |  |  |  |  |  |  |  |  |  |
| **2001–2003** |  |  |  |  |  |  |  |  |  |  |  |  |
| Not clustered and not neighbour | 42 | 9.3 | 1,253 | 69.8 | 1,237 | 74.2 | 1,089 | 91.4 | 110 | 23.6 | 3,731 | 67.0 |
| Not significant cluster neighbour | 0 | 0.0 | 8 | 0.4 | 18 | 1.1 | 27 | 2.3 | 0 | 0.0 | 53 | 1.0 |
| Not significant cluster | 0 | 0.0 | 2 | 0.1 | 5 | 0.3 | 13 | 1.1 | 0 | 0.0 | 20 | 0.4 |
| Significant cluster neighbour | 67 | 14.9 | 166 | 9.3 | 212 | 12.7 | 46 | 3.9 | 62 | 13.3 | 553 | 9.9 |
| Significant cluster | 341 | 75.8 | 365 | 20.3 | 196 | 11.8 | 16 | 1.3 | 295 | 63.2 | 1,213 | 21.8 |
| **2003–2005** |  |  |  |  |  |  |  |  |  |  |  |  |
| Not clustered and not neighbour | 34 | 7.6 | 1,014 | 56.5 | 1,257 | 75.4 | 1,090 | 91.5 | 70 | 15.0 | 3,465 | 62.2 |
| Not significant cluster neighbour | 13 | 2.9 | 8 | 0.4 | 44 | 2.6 | 23 | 1.9 | 7 | 1.5 | 95 | 1.7 |
| Not significant cluster | 10 | 2.2 | 1 | 0.1 | 14 | 0.8 | 10 | 0.8 | 1 | 0.2 | 36 | 0.6 |
| Significant cluster neighbour | 66 | 14.7 | 206 | 11.5 | 161 | 9.7 | 36 | 3.0 | 63 | 13.5 | 532 | 9.6 |
| Significant cluster | 327 | 72.7 | 565 | 31.5 | 192 | 11.5 | 32 | 2.7 | 326 | 69.8 | 1,442 | 25.9 |
| **2005–2007** |  |  |  |  |  |  |  |  |  |  |  |  |
| Not clustered and not neighbour | 39 | 8.7 | 1,083 | 60.4 | 1,256 | 75.3 | 1,086 | 91.2 | 103 | 22.1 | 3,567 | 64.0 |
| Not significant cluster neighbour | 0 | 0.0 | 6 | 0.3 | 35 | 2.1 | 35 | 2.9 | 4 | 0.9 | 80 | 1.4 |
| Not significant cluster | 0 | 0.0 | 1 | 0.1 | 13 | 0.8 | 9 | 0.8 | 1 | 0.2 | 24 | 0.4 |
| Significant cluster neighbour | 73 | 16.2 | 193 | 10.8 | 176 | 10.6 | 42 | 3.5 | 67 | 14.3 | 551 | 9.9 |
| Significant cluster | 338 | 75.1 | 511 | 28.5 | 188 | 11.3 | 19 | 1.6 | 292 | 62.5 | 1,348 | 24.2 |
| **2007–2009** |  |  |  |  |  |  |  |  |  |  |  |  |
| Not clustered and not neighbour | 33 | 7.3 | 1,092 | 60.9 | 1,322 | 79.3 | 1,054 | 88.5 | 88 | 18.8 | 3,589 | 64.4 |
| Not significant cluster neighbour | 0 | 0.0 | 35 | 2.0 | 23 | 1.4 | 51 | 4.3 | 2 | 0.4 | 111 | 2.0 |
| Not significant cluster | 0 | 0.0 | 5 | 0.3 | 4 | 0.2 | 32 | 2.7 | 1 | 0.2 | 42 | 0.8 |
| Significant cluster neighbour | 76 | 16.9 | 154 | 8.6 | 132 | 7.9 | 40 | 3.4 | 67 | 14.3 | 469 | 8.4 |
| Significant cluster | 341 | 75.8 | 508 | 28.3 | 187 | 11.2 | 14 | 1.2 | 309 | 66.2 | 1,359 | 24.4 |
| **2009–2011** |  |  |  |  |  |  |  |  |  |  |  |  |
| Not clustered and not neighbour | 52 | 11.6 | 1,085 | 60.5 | 1,319 | 79.1 | 1,118 | 93.9 | 90 | 19.3 | 3,664 | 65.8 |
| Not significant cluster neighbour | 0 | 0.0 | 27 | 1.5 | 42 | 2.5 | 19 | 1.6 | 0 | 0.0 | 88 | 1.6 |
| Not significant cluster | 0 | 0.0 | 4 | 0.2 | 10 | 0.6 | 4 | 0.3 | 0 | 0.0 | 18 | 0.3 |
| Significant cluster neighbour | 74 | 16.4 | 174 | 9.7 | 112 | 6.7 | 35 | 2.9 | 92 | 19.7 | 487 | 8.7 |
| Significant cluster | 324 | 72.0 | 504 | 28.1 | 185 | 11.1 | 15 | 1.3 | 285 | 61.0 | 1,313 | 23.6 |
| **2011–2013** |  |  |  |  |  |  |  |  |  |  |  |  |
| Not clustered and not neighbour | 47 | 10.4 | 1,148 | 64.0 | 1,384 | 83.0 | 1,100 | 92.4 | 70 | 15.0 | 3,749 | 67.3 |
| Not significant cluster neighbour | 3 | 0.7 | 16 | 0.9 | 27 | 1.6 | 21 | 1.8 | 1 | 0.2 | 68 | 1.2 |
| Not significant cluster | 5 | 1.1 | 3 | 0.2 | 11 | 0.7 | 11 | 0.9 | 0 | 0.0 | 30 | 0.5 |
| Significant cluster neighbour | 65 | 14.4 | 153 | 8.5 | 113 | 6.8 | 34 | 2.9 | 85 | 18.2 | 450 | 8.1 |
| Significant cluster | 330 | 73.3 | 474 | 26.4 | 133 | 8.0 | 25 | 2.1 | 311 | 66.6 | 1,273 | 22.9 |
| **2013–2015** |  |  |  |  |  |  |  |  |  |  |  |  |
| Not clustered and not neighbour | 85 | 18.9 | 982 | 54.7 | 1,402 | 84.1 | 1,133 | 95.1 | 158 | 33.8 | 3,760 | 67.5 |
| Not significant cluster neighbour | 0 | 0.0 | 9 | 0.5 | 4 | 0.2 | 37 | 3.1 | 0 | 0.0 | 50 | 0.9 |
| Not significant cluster | 0 | 0.0 | 5 | 0.3 | 0 | 0.0 | 12 | 1.0 | 0 | 0.0 | 17 | 0.3 |
| Significant cluster neighbour | 78 | 17.3 | 188 | 10.5 | 126 | 7.6 | 8 | 0.7 | 75 | 16.1 | 475 | 8.5 |
| Significant cluster | 287 | 63.8 | 610 | 34.0 | 136 | 8.2 | 1 | 0.1 | 234 | 50.1 | 1,268 | 22.8 |
| **2015–2017** |  |  |  |  |  |  |  |  |  |  |  |  |
| Not clustered and not neighbour | 80 | 17.8 | 845 | 47.1 | 1,440 | 86.3 | 1,154 | 96.9 | 157 | 33.6 | 3,676 | 66.0 |
| Not significant cluster neighbour | 6 | 1.3 | 0 | 0.0 | 8 | 0.5 | 19 | 1.6 | 0 | 0.0 | 33 | 0.6 |
| Not significant cluster | 2 | 0.4 | 0 | 0.0 | 2 | 0.1 | 7 | 0.6 | 0 | 0.0 | 11 | 0.2 |
| Significant cluster neighbour | 76 | 16.9 | 204 | 11.4 | 84 | 5.0 | 9 | 0.8 | 77 | 16.5 | 450 | 8.1 |
| Significant cluster | 286 | 63.6 | 745 | 41.5 | 134 | 8.0 | 2 | 0.2 | 233 | 49.9 | 1,400 | 25.1 |
| **2017–2019** |  |  |  |  |  |  |  |  |  |  |  |  |
| Not clustered and not neighbour | 67 | 14.9 | 953 | 53.1 | 1,426 | 85.5 | 1,172 | 98.4 | 138 | 29.6 | 3,756 | 67.4 |
| Not significant cluster neighbour | 4 | 0.9 | 8 | 0.4 | 22 | 1.3 | 14 | 1.2 | 0 | 0.0 | 48 | 0.9 |
| Not significant cluster | 1 | 0.2 | 3 | 0.2 | 3 | 0.2 | 2 | 0.2 | 0 | 0.0 | 9 | 0.2 |
| Significant cluster neighbour | 88 | 19.6 | 198 | 11.0 | 78 | 4.7 | 3 | 0.3 | 68 | 14.6 | 435 | 7.8 |
| Significant cluster | 290 | 64.4 | 632 | 35.2 | 139 | 8.3 | 0 | 0.0 | 261 | 55.9 | 1,322 | 23.7 |
| **2019–2021** |  |  |  |  |  |  |  |  |  |  |  |  |
| Not clustered and not neighbour | 65 | 14.4 | 1,133 | 63.2 | 1,471 | 88.2 | 1,155 | 97.0 | 112 | 24.0 | 3,936 | 70.7 |
| Not significant cluster neighbour | 3 | 0.7 | 0 | 0.0 | 14 | 0.8 | 0 | 0.0 | 10 | 2.1 | 27 | 0.5 |
| Not significant cluster | 1 | 0.2 | 0 | 0.0 | 31 | 1.9 | 0 | 0.0 | 1 | 0.2 | 33 | 0.6 |
| Significant cluster neighbour | 81 | 18.0 | 138 | 7.7 | 84 | 5.0 | 18 | 1.5 | 81 | 17.3 | 402 | 7.2 |
| Significant cluster | 300 | 66.7 | 523 | 29.2 | 68 | 4.1 | 18 | 1.5 | 263 | 56.3 | 1,172 | 21.0 |
| **2021–2023** |  |  |  |  |  |  |  |  |  |  |  |  |
| Not clustered and not neighbour | 126 | 28.0 | 1,341 | 74.7 | 1,460 | 87.5 | 1,138 | 95.5 | 135 | 28.9 | 4,200 | 75.4 |
| Not significant cluster neighbour | 9 | 2.0 | 7 | 0.4 | 22 | 1.3 | 27 | 2.3 | 9 | 1.9 | 74 | 1.3 |
| Not significant cluster | 19 | 4.2 | 1 | 0.1 | 7 | 0.4 | 5 | 0.4 | 3 | 0.6 | 35 | 0.6 |
| Significant cluster neighbour | 62 | 13.8 | 97 | 5.4 | 75 | 4.5 | 17 | 1.4 | 71 | 15.2 | 322 | 5.8 |
| Significant cluster | 234 | 52.0 | 348 | 19.4 | 104 | 6.2 | 4 | 0.3 | 249 | 53.3 | 939 | 16.9 |
| **Ranking – comparison** |  |  |  |  |  |  |  |  |  |  |  |  |
| **2001–2003** |  |  |  |  |  |  |  |  |  |  |  |  |
| Not in cluster | - |  | - |  | - |  | - |  | - |  | - |  |
| Neighbour of cluster | - |  | - |  | - |  | - |  | - |  | - |  |
| Left a cluster | - |  | - |  | - |  | - |  | - |  | - |  |
| Joined a cluster | - |  | - |  | - |  | - |  | - |  | - |  |
| Always been in cluster | - |  | - |  | - |  | - |  | - |  | - |  |
| **2003–2005** |  |  |  |  |  |  |  |  |  |  |  |  |
| Not in cluster | 22 | 4.9 | 961 | 53.6 | 1,162 | 69.7 | 1,070 | 89.8 | 69 | 14.8 | 3,284 | 59.0 |
| Neighbour of cluster | 41 | 9.1 | 72 | 4.0 | 84 | 5.0 | 25 | 2.1 | 53 | 11.3 | 275 | 4.9 |
| Left a cluster | 48 | 10.7 | 78 | 4.3 | 160 | 9.6 | 35 | 2.9 | 2 | 0.4 | 323 | 5.8 |
| Joined a cluster | 28 | 6.2 | 351 | 19.6 | 119 | 7.1 | 48 | 4.0 | 49 | 10.5 | 595 | 10.7 |
| Always been in cluster | 311 | 69.1 | 332 | 18.5 | 143 | 8.6 | 13 | 1.1 | 294 | 63.0 | 1,093 | 19.6 |
| **2005–2007** |  |  |  |  |  |  |  |  |  |  |  |  |
| Not in cluster | 21 | 4.7 | 887 | 49.4 | 1,137 | 68.2 | 1,041 | 87.4 | 69 | 14.8 | 3,155 | 56.6 |
| Neighbour of cluster | 38 | 8.4 | 53 | 3.0 | 62 | 3.7 | 14 | 1.2 | 51 | 10.9 | 218 | 3.9 |
| Left a cluster | 33 | 7.3 | 65 | 3.6 | 131 | 7.9 | 48 | 4.0 | 10 | 2.1 | 287 | 5.2 |
| Joined a cluster | 48 | 10.7 | 472 | 26.3 | 206 | 12.4 | 82 | 6.9 | 47 | 10.1 | 855 | 15.4 |
| Always been in cluster | 310 | 68.9 | 317 | 17.7 | 132 | 7.9 | 6 | 0.5 | 290 | 62.1 | 1,055 | 18.9 |
| **2007–2009** |  |  |  |  |  |  |  |  |  |  |  |  |
| Not in cluster | 14 | 3.1 | 878 | 48.9 | 1,123 | 67.3 | 1,010 | 84.8 | 36 | 7.7 | 3,061 | 55.0 |
| Neighbour of cluster | 24 | 5.3 | 50 | 2.8 | 40 | 2.4 | 12 | 1.0 | 26 | 5.6 | 152 | 2.7 |
| Left a cluster | 46 | 10.2 | 60 | 3.3 | 158 | 9.5 | 43 | 3.6 | 43 | 9.2 | 350 | 6.3 |
| Joined a cluster | 69 | 15.3 | 489 | 27.3 | 224 | 13.4 | 124 | 10.4 | 100 | 21.4 | 1,006 | 18.1 |
| Always been in cluster | 297 | 66.0 | 317 | 17.7 | 123 | 7.4 | 2 | 0.2 | 262 | 56.1 | 1,001 | 18.0 |
| **2009–2011** |  |  |  |  |  |  |  |  |  |  |  |  |
| Not in cluster | 10 | 2.2 | 855 | 47.7 | 1,099 | 65.9 | 1,009 | 84.7 | 30 | 6.4 | 3,003 | 53.9 |
| Neighbour of cluster | 18 | 4.0 | 43 | 2.4 | 25 | 1.5 | 9 | 0.8 | 10 | 2.1 | 105 | 1.9 |
| Left a cluster | 70 | 15.6 | 59 | 3.3 | 154 | 9.2 | 50 | 4.2 | 90 | 19.3 | 423 | 7.6 |
| Joined a cluster | 71 | 15.8 | 528 | 29.4 | 272 | 16.3 | 122 | 10.2 | 147 | 31.5 | 1,140 | 20.5 |
| Always been in cluster | 281 | 62.4 | 309 | 17.2 | 118 | 7.1 | 1 | 0.1 | 190 | 40.7 | 899 | 16.1 |
| **2011–2013** |  |  |  |  |  |  |  |  |  |  |  |  |
| Not in cluster | 10 | 2.2 | 838 | 46.7 | 1,099 | 65.9 | 1,001 | 84.0 | 6 | 1.3 | 2,954 | 53.0 |
| Neighbour of cluster | 16 | 3.6 | 28 | 1.6 | 16 | 1.0 | 8 | 0.7 | 8 | 1.7 | 76 | 1.4 |
| Left a cluster | 64 | 14.2 | 55 | 3.1 | 196 | 11.8 | 30 | 2.5 | 95 | 20.3 | 440 | 7.9 |
| Joined a cluster | 85 | 18.9 | 566 | 31.5 | 269 | 16.1 | 151 | 12.7 | 172 | 36.8 | 1,243 | 22.3 |
| Always been in cluster | 275 | 61.1 | 307 | 17.1 | 88 | 5.3 | 1 | 0.1 | 186 | 39.8 | 857 | 15.4 |
| **2013–2015** |  |  |  |  |  |  |  |  |  |  |  |  |
| Not in cluster | 7 | 1.6 | 821 | 45.8 | 1,087 | 65.2 | 986 | 82.8 | 4 | 0.9 | 2,905 | 52.2 |
| Neighbour of cluster | 15 | 3.3 | 27 | 1.5 | 13 | 0.8 | 6 | 0.5 | 5 | 1.1 | 66 | 1.2 |
| Left a cluster | 78 | 17.3 | 34 | 1.9 | 204 | 12.2 | 49 | 4.1 | 126 | 27.0 | 491 | 8.8 |
| Joined a cluster | 98 | 21.8 | 605 | 33.7 | 300 | 18.0 | 149 | 12.5 | 202 | 43.3 | 1,354 | 24.3 |
| Always been in cluster | 252 | 56.0 | 307 | 17.1 | 64 | 3.8 | 1 | 0.1 | 130 | 27.8 | 754 | 13.5 |
| **2015–2017** |  |  |  |  |  |  |  |  |  |  |  |  |
| Not in cluster | 6 | 1.3 | 741 | 41.3 | 1,075 | 64.4 | 978 | 82.1 | 4 | 0.9 | 2,804 | 50.3 |
| Neighbour of cluster | 13 | 2.9 | 23 | 1.3 | 8 | 0.5 | 5 | 0.4 | 3 | 0.6 | 52 | 0.9 |
| Left a cluster | 78 | 17.3 | 27 | 1.5 | 209 | 12.5 | 57 | 4.8 | 126 | 27.0 | 497 | 8.9 |
| Joined a cluster | 109 | 24.2 | 696 | 38.8 | 317 | 19.0 | 151 | 12.7 | 209 | 44.8 | 1,482 | 26.6 |
| Always been in cluster | 244 | 54.2 | 307 | 17.1 | 59 | 3.5 | 0 | 0.0 | 125 | 26.8 | 735 | 13.2 |
| **2017–2019** |  |  |  |  |  |  |  |  |  |  |  |  |
| Not in cluster | 1 | 0.2 | 635 | 35.4 | 1,057 | 63.4 | 978 | 82.1 | 3 | 0.6 | 2,674 | 48.0 |
| Neighbour of cluster | 4 | 0.9 | 3 | 0.2 | 8 | 0.5 | 5 | 0.4 | 2 | 0.4 | 22 | 0.4 |
| Left a cluster | 87 | 19.3 | 83 | 4.6 | 230 | 13.8 | 51 | 4.3 | 91 | 19.5 | 542 | 9.7 |
| Joined a cluster | 152 | 33.8 | 814 | 45.4 | 356 | 21.3 | 157 | 13.2 | 262 | 56.1 | 1,741 | 31.3 |
| Always been in cluster | 206 | 45.8 | 259 | 14.4 | 17 | 1.0 | 0 | 0.0 | 109 | 23.3 | 591 | 10.6 |
| **2019–2021** |  |  |  |  |  |  |  |  |  |  |  |  |
| Not in cluster | 1 | 0.2 | 634 | 35.3 | 1,039 | 62.3 | 962 | 80.8 | 2 | 0.4 | 2,638 | 47.4 |
| Neighbour of cluster | 2 | 0.4 | 3 | 0.2 | 10 | 0.6 | 3 | 0.3 | 0 | 0.0 | 18 | 0.3 |
| Left a cluster | 70 | 15.6 | 77 | 4.3 | 181 | 10.9 | 52 | 4.4 | 102 | 21.8 | 482 | 8.7 |
| Joined a cluster | 200 | 44.4 | 821 | 45.8 | 421 | 25.2 | 174 | 14.6 | 274 | 58.7 | 1,890 | 33.9 |
| Always been in cluster | 177 | 39.3 | 259 | 14.4 | 17 | 1.0 | 0 | 0.0 | 89 | 19.1 | 542 | 9.7 |
| **2021–2023** |  |  |  |  |  |  |  |  |  |  |  |  |
| Not in cluster | 0 | 0.0 | 634 | 35.3 | 1,030 | 61.8 | 936 | 78.6 | 2 | 0.4 | 2,602 | 46.7 |
| Neighbour of cluster | 1 | 0.2 | 2 | 0.1 | 10 | 0.6 | 3 | 0.3 | 0 | 0.0 | 16 | 0.3 |
| Left a cluster | 79 | 17.6 | 97 | 5.4 | 146 | 8.8 | 48 | 4.0 | 68 | 14.6 | 438 | 7.9 |
| Joined a cluster | 209 | 46.4 | 807 | 45.0 | 465 | 27.9 | 204 | 17.1 | 319 | 68.3 | 2,004 | 36.0 |
| Always been in cluster | 161 | 35.8 | 254 | 14.2 | 17 | 1.0 | 0 | 0.0 | 78 | 16.7 | 510 | 9.2 |

N: Number, %: Percentage.
