# Supplementary material for: Persistent and Emerging High‐Risk Clusters of Leprosy Detection in Brazil: A Nationwide Spatiotemporal Analysis, 2001–2023
Source: Trop Med Int Health. 2026 Feb 16;31(4):532–46. doi: 10.1111/tmi.70104 (PMC13050618; doi:10.1111/tmi.70104)
Supplement: Supplementary file 3 — Table S2: Classification of epidemiological and operational groups in the 2019–2022 Strategy in relation to clusters and their temporal evolution, Brazil, 2001–2023. [file TMI-31-532-s003.docx]

**Supplements – Table 2:** Classification of epidemiological and operational groups in the 2019–2022 Strategy in relation to clusters and their temporal evolution, Brazil, 2001–2023

| **Municipality classification** | **No cases** | | **Subgroup 2.1** | | **Subgroup 2.2** | | **Subgroup 2.3** | | **Subgroup 2.4** | | **Subgroup 3.1** | | **Subgroup 3.2** | | **Subgroup 3.3** | | **Subgroup 3.4** | |
| --- | --- | --- | --- | --- | --- | --- | --- | --- | --- | --- | --- | --- | --- | --- | --- | --- | --- | --- |
|  | **N** | **%** | **N** | **%** | **N** | **%** | **N** | **%** | **N** | **%** | **N** | **%** | **N** | **%** | **N** | **%** | **N** | **%** |
| **Number of municipalities** | **1,118** | **20.1** | **435** | **7.8** | **213** | **3.8** | **458** | **8.2** | **1,122** | **20.1** | **402** | **7.2** | **422** | **7.6** | **763** | **13.7** | **631** | **11.3** |
| **Ranking** |  |  |  |  |  |  |  |  |  |  |  |  |  |  |  |  |  |  |
| **2001–2003** |  |  |  |  |  |  |  |  |  |  |  |  |  |  |  |  |  |  |
| Not clustered and not neighbour | 1,034 | 92.5 | 359 | 82.5 | 182 | 85.4 | 376 | 82.1 | 929 | 82.8 | 173 | 43.0 | 134 | 31.8 | 264 | 34.6 | 276 | 43.7 |
| Not significant cluster neighbour | 9 | 2.0 | 6 | 1.4 | 4 | 1.9 | 10 | 2.2 | 12 | 1.1 | 1 | 0.2 | 1 | 0.2 | 7 | 0.9 | 3 | 0.5 |
| Not significant cluster | 2 | 0.4 | 0 | 0.0 | 2 | 0.9 | 1 | 0.2 | 4 | 0.4 | 0 | 0.0 | 1 | 0.2 | 7 | 0.9 | 3 | 0.5 |
| Significant cluster neighbour | 51 | 11.3 | 44 | 10.1 | 17 | 8.0 | 44 | 9.6 | 102 | 9.1 | 47 | 11.7 | 62 | 14.7 | 104 | 13.6 | 82 | 13.0 |
| Significant cluster | 22 | 4.9 | 26 | 6.0 | 8 | 3.8 | 27 | 5.9 | 75 | 6.7 | 181 | 45.0 | 224 | 53.1 | 381 | 49.9 | 267 | 42.3 |
| **2003–2005** |  |  |  |  |  |  |  |  |  |  |  |  |  |  |  |  |  |  |
| Not clustered and not neighbour | 1,028 | 91.9 | 321 | 73.8 | 177 | 83.1 | 360 | 78.6 | 893 | 79.6 | 122 | 30.3 | 124 | 29.4 | 212 | 27.8 | 225 | 35.7 |
| Not significant cluster neighbour | 9 | 2.0 | 4 | 0.9 | 4 | 1.9 | 10 | 2.2 | 24 | 2.1 | 3 | 0.7 | 5 | 1.2 | 21 | 2.8 | 15 | 2.4 |
| Not significant cluster | 1 | 0.2 | 3 | 0.7 | 0 | 0.0 | 2 | 0.4 | 4 | 0.4 | 3 | 0.7 | 1 | 0.2 | 16 | 2.1 | 6 | 1.0 |
| Significant cluster neighbour | 45 | 10.0 | 51 | 11.7 | 16 | 7.5 | 53 | 11.6 | 91 | 8.1 | 58 | 14.4 | 58 | 13.7 | 87 | 11.4 | 73 | 11.6 |
| Significant cluster | 35 | 7.8 | 56 | 12.9 | 16 | 7.5 | 33 | 7.2 | 110 | 9.8 | 216 | 53.7 | 234 | 55.5 | 427 | 56.0 | 312 | 49.4 |
| **2005–2007** |  |  |  |  |  |  |  |  |  |  |  |  |  |  |  |  |  |  |
| Not clustered and not neighbour | 1,032 | 92.3 | 331 | 76.1 | 181 | 85.0 | 372 | 81.2 | 906 | 80.7 | 142 | 35.3 | 134 | 31.8 | 226 | 29.6 | 239 | 37.9 |
| Not significant cluster neighbour | 13 | 2.9 | 8 | 1.8 | 4 | 1.9 | 11 | 2.4 | 25 | 2.2 | 3 | 0.7 | 1 | 0.2 | 6 | 0.8 | 9 | 1.4 |
| Not significant cluster | 1 | 0.2 | 0 | 0.0 | 2 | 0.9 | 1 | 0.2 | 2 | 0.2 | 5 | 1.2 | 0 | 0.0 | 8 | 1.0 | 5 | 0.8 |
| Significant cluster neighbour | 39 | 8.7 | 49 | 11.3 | 15 | 7.0 | 50 | 10.9 | 99 | 8.8 | 55 | 13.7 | 58 | 13.7 | 110 | 14.4 | 76 | 12.0 |
| Significant cluster | 33 | 7.3 | 47 | 10.8 | 11 | 5.2 | 24 | 5.2 | 90 | 8.0 | 197 | 49.0 | 229 | 54.3 | 413 | 54.1 | 302 | 47.9 |
| **2007–2009** |  |  |  |  |  |  |  |  |  |  |  |  |  |  |  |  |  |  |
| Not clustered and not neighbour | 1,050 | 93.9 | 334 | 76.8 | 179 | 84.0 | 379 | 82.8 | 900 | 80.2 | 145 | 36.1 | 138 | 32.7 | 235 | 30.8 | 225 | 35.7 |
| Not significant cluster neighbour | 6 | 1.3 | 16 | 3.7 | 6 | 2.8 | 17 | 3.7 | 40 | 3.6 | 4 | 1.0 | 2 | 0.5 | 11 | 1.4 | 9 | 1.4 |
| Not significant cluster | 1 | 0.2 | 0 | 0.0 | 0 | 0.0 | 0 | 0.0 | 12 | 1.1 | 4 | 1.0 | 3 | 0.7 | 9 | 1.2 | 13 | 2.1 |
| Significant cluster neighbour | 31 | 6.9 | 35 | 8.0 | 16 | 7.5 | 38 | 8.3 | 79 | 7.0 | 47 | 11.7 | 49 | 11.6 | 98 | 12.8 | 76 | 12.0 |
| Significant cluster | 30 | 6.7 | 50 | 11.5 | 12 | 5.6 | 24 | 5.2 | 91 | 8.1 | 202 | 50.2 | 230 | 54.5 | 410 | 53.7 | 308 | 48.8 |
| **2009–2011** |  |  |  |  |  |  |  |  |  |  |  |  |  |  |  |  |  |  |
| Not clustered and not neighbour | 1,060 | 94.8 | 342 | 78.6 | 187 | 87.8 | 382 | 83.4 | 933 | 83.2 | 139 | 34.6 | 130 | 30.8 | 243 | 31.8 | 244 | 38.7 |
| Not significant cluster neighbour | 9 | 2.0 | 10 | 2.3 | 2 | 0.9 | 13 | 2.8 | 22 | 2.0 | 6 | 1.5 | 5 | 1.2 | 11 | 1.4 | 10 | 1.6 |
| Not significant cluster | 0 | 0.0 | 0 | 0.0 | 0 | 0.0 | 1 | 0.2 | 1 | 0.1 | 3 | 0.7 | 3 | 0.7 | 5 | 0.7 | 5 | 0.8 |
| Significant cluster neighbour | 23 | 5.1 | 36 | 8.3 | 13 | 6.1 | 36 | 7.9 | 82 | 7.3 | 51 | 12.7 | 60 | 14.2 | 106 | 13.9 | 80 | 12.7 |
| Significant cluster | 26 | 5.8 | 47 | 10.8 | 11 | 5.2 | 26 | 5.7 | 84 | 7.5 | 203 | 50.5 | 224 | 53.1 | 398 | 52.2 | 292 | 46.3 |
| **2011–2013** |  |  |  |  |  |  |  |  |  |  |  |  |  |  |  |  |  |  |
| Not clustered and not neighbour | 1,062 | 95.0 | 354 | 81.4 | 188 | 88.3 | 403 | 88.0 | 961 | 85.7 | 146 | 36.3 | 145 | 34.4 | 242 | 31.7 | 244 | 38.7 |
| Not significant cluster neighbour | 7 | 1.6 | 8 | 1.8 | 4 | 1.9 | 11 | 2.4 | 14 | 1.2 | 1 | 0.2 | 6 | 1.4 | 10 | 1.3 | 7 | 1.1 |
| Not significant cluster | 0 | 0.0 | 2 | 0.5 | 1 | 0.5 | 1 | 0.2 | 2 | 0.2 | 4 | 1.0 | 2 | 0.5 | 11 | 1.4 | 7 | 1.1 |
| Significant cluster neighbour | 30 | 6.7 | 33 | 7.6 | 11 | 5.2 | 27 | 5.9 | 71 | 6.3 | 48 | 11.9 | 48 | 11.4 | 94 | 12.3 | 88 | 13.9 |
| Significant cluster | 19 | 4.2 | 38 | 8.7 | 9 | 4.2 | 16 | 3.5 | 74 | 6.6 | 203 | 50.5 | 221 | 52.4 | 406 | 53.2 | 285 | 45.2 |
| **2013–2015** |  |  |  |  |  |  |  |  |  |  |  |  |  |  |  |  |  |  |
| Not clustered and not neighbour | 1,052 | 94.1 | 333 | 76.6 | 185 | 86.9 | 401 | 87.6 | 958 | 85.4 | 138 | 34.3 | 142 | 33.6 | 267 | 35.0 | 281 | 44.5 |
| Not significant cluster neighbour | 8 | 1.8 | 4 | 0.9 | 1 | 0.5 | 4 | 0.9 | 17 | 1.5 | 0 | 0.0 | 1 | 0.2 | 6 | 0.8 | 9 | 1.4 |
| Not significant cluster | 0 | 0.0 | 1 | 0.2 | 1 | 0.5 | 1 | 0.2 | 2 | 0.2 | 2 | 0.5 | 2 | 0.5 | 5 | 0.7 | 3 | 0.5 |
| Significant cluster neighbour | 28 | 6.2 | 40 | 9.2 | 13 | 6.1 | 29 | 6.3 | 70 | 6.2 | 46 | 11.4 | 48 | 11.4 | 113 | 14.8 | 87 | 13.8 |
| Significant cluster | 30 | 6.7 | 57 | 13.1 | 13 | 6.1 | 23 | 5.0 | 75 | 6.7 | 216 | 53.7 | 229 | 54.3 | 372 | 48.8 | 251 | 39.8 |
| **2015–2017** |  |  |  |  |  |  |  |  |  |  |  |  |  |  |  |  |  |  |
| Not clustered and not neighbour | 1,042 | 93.2 | 317 | 72.9 | 183 | 85.9 | 398 | 86.9 | 941 | 83.9 | 117 | 29.1 | 137 | 32.5 | 262 | 34.3 | 275 | 43.6 |
| Not significant cluster neighbour | 5 | 1.1 | 1 | 0.2 | 0 | 0.0 | 5 | 1.1 | 8 | 0.7 | 2 | 0.5 | 0 | 0.0 | 8 | 1.0 | 4 | 0.6 |
| Not significant cluster | 0 | 0.0 | 0 | 0.0 | 0 | 0.0 | 0 | 0.0 | 0 | 0.0 | 1 | 0.2 | 1 | 0.2 | 5 | 0.7 | 4 | 0.6 |
| Significant cluster neighbour | 32 | 7.1 | 32 | 7.4 | 12 | 5.6 | 25 | 5.5 | 73 | 6.5 | 53 | 13.2 | 43 | 10.2 | 99 | 13.0 | 81 | 12.8 |
| Significant cluster | 39 | 8.7 | 85 | 19.5 | 18 | 8.5 | 30 | 6.6 | 100 | 8.9 | 229 | 57.0 | 241 | 57.1 | 389 | 51.0 | 267 | 42.3 |
| **2017–2019** |  |  |  |  |  |  |  |  |  |  |  |  |  |  |  |  |  |  |
| Not clustered and not neighbour | 1,059 | 94.7 | 336 | 77.2 | 188 | 88.3 | 400 | 87.3 | 959 | 85.5 | 132 | 32.8 | 122 | 28.9 | 267 | 35.0 | 290 | 46.0 |
| Not significant cluster neighbour | 4 | 0.9 | 3 | 0.7 | 2 | 0.9 | 8 | 1.7 | 10 | 0.9 | 1 | 0.2 | 3 | 0.7 | 12 | 1.6 | 5 | 0.8 |
| Not significant cluster | 0 | 0.0 | 0 | 0.0 | 0 | 0.0 | 0 | 0.0 | 0 | 0.0 | 1 | 0.2 | 0 | 0.0 | 7 | 0.9 | 1 | 0.2 |
| Significant cluster neighbour | 23 | 5.1 | 43 | 9.9 | 11 | 5.2 | 21 | 4.6 | 65 | 5.8 | 48 | 11.9 | 55 | 13.0 | 93 | 12.2 | 76 | 12.0 |
| Significant cluster | 32 | 7.1 | 53 | 12.2 | 12 | 5.6 | 29 | 6.3 | 88 | 7.8 | 220 | 54.7 | 242 | 57.3 | 384 | 50.3 | 259 | 41.0 |
| **2019–2021** |  |  |  |  |  |  |  |  |  |  |  |  |  |  |  |  |  |  |
| Not clustered and not neighbour | 1,067 | 95.4 | 365 | 83.9 | 195 | 91.5 | 428 | 93.4 | 990 | 88.2 | 156 | 38.8 | 135 | 32.0 | 298 | 39.1 | 298 | 47.2 |
| Not significant cluster neighbour | 2 | 0.4 | 1 | 0.2 | 2 | 0.9 | 3 | 0.7 | 5 | 0.4 | 1 | 0.2 | 3 | 0.7 | 6 | 0.8 | 4 | 0.6 |
| Not significant cluster | 5 | 1.1 | 1 | 0.2 | 1 | 0.5 | 2 | 0.4 | 10 | 0.9 | 1 | 0.2 | 1 | 0.2 | 9 | 1.2 | 3 | 0.5 |
| Significant cluster neighbour | 26 | 5.8 | 32 | 7.4 | 7 | 3.3 | 13 | 2.8 | 46 | 4.1 | 44 | 10.9 | 54 | 12.8 | 95 | 12.5 | 85 | 13.5 |
| Significant cluster | 18 | 4.0 | 36 | 8.3 | 8 | 3.8 | 12 | 2.6 | 71 | 6.3 | 200 | 49.8 | 229 | 54.3 | 355 | 46.5 | 241 | 38.2 |
| **2021–2023** |  |  |  |  |  |  |  |  |  |  |  |  |  |  |  |  |  |  |
| Not clustered and not neighbour | 1,071 | 95.8 | 389 | 89.4 | 194 | 91.1 | 423 | 92.4 | 1,018 | 90.7 | 216 | 53.7 | 186 | 44.1 | 352 | 46.1 | 347 | 55.0 |
| Not significant cluster neighbour | 9 | 2.0 | 5 | 1.1 | 2 | 0.9 | 7 | 1.5 | 19 | 1.7 | 4 | 1.0 | 4 | 0.9 | 9 | 1.2 | 15 | 2.4 |
| Not significant cluster | 0 | 0.0 | 2 | 0.5 | 1 | 0.5 | 1 | 0.2 | 5 | 0.4 | 2 | 0.5 | 8 | 1.9 | 7 | 0.9 | 9 | 1.4 |
| Significant cluster neighbour | 21 | 4.7 | 14 | 3.2 | 8 | 3.8 | 13 | 2.8 | 37 | 3.3 | 26 | 6.5 | 52 | 12.3 | 91 | 11.9 | 60 | 9.5 |
| Significant cluster | 17 | 3.8 | 25 | 5.7 | 8 | 3.8 | 14 | 3.1 | 43 | 3.8 | 154 | 38.3 | 172 | 40.8 | 304 | 39.8 | 200 | 31.7 |
| **Ranking – comparison** |  |  |  |  |  |  |  |  |  |  |  |  |  |  |  |  |  |  |
| **2001–2003** |  |  |  |  |  |  |  |  |  |  |  |  |  |  |  |  |  |  |
| Not in cluster | - |  | - |  | - |  | - |  | - |  | - | - | - | - | - | - | - | - |
| Neighbour of cluster | - |  | - |  | - |  | - |  | - |  | - | - | - | - | - | - | - | - |
| Left a cluster | - |  | - |  | - |  | - |  | - |  | - | - | - | - | - | - | - | - |
| Joined a cluster | - |  | - |  | - |  | - |  | - |  | - | - | - | - | - | - | - | - |
| Always been in cluster | - |  | - |  | - |  | - |  | - |  | - | - | - | - | - | - | - | - |
| **2003–2005** |  |  |  |  |  |  |  |  |  |  |  |  |  |  |  |  |  |  |
| Not in cluster | 992 | 88.7 | 307 | 70.6 | 169 | 79.3 | 346 | 75.5 | 855 | 76.2 | 116 | 28.9 | 106 | 25.1 | 187 | 24.5 | 203 | 32.2 |
| Neighbour of cluster | 15 | 3.3 | 24 | 5.5 | 9 | 4.2 | 30 | 6.6 | 38 | 3.4 | 30 | 7.5 | 35 | 8.3 | 52 | 6.8 | 42 | 6.7 |
| Left a cluster | 46 | 10.2 | 17 | 3.9 | 9 | 4.2 | 26 | 5.7 | 68 | 6.1 | 16 | 4.0 | 33 | 7.8 | 60 | 7.9 | 48 | 7.6 |
| Joined a cluster | 53 | 11.8 | 64 | 14.7 | 19 | 8.9 | 38 | 8.3 | 98 | 8.7 | 70 | 17.4 | 43 | 10.2 | 110 | 14.4 | 99 | 15.7 |
| Always been in cluster | 12 | 2.7 | 23 | 5.3 | 7 | 3.3 | 18 | 3.9 | 63 | 5.6 | 170 | 42.3 | 205 | 48.6 | 354 | 46.4 | 239 | 37.9 |
| **2005–2007** |  |  |  |  |  |  |  |  |  |  |  |  |  |  |  |  |  |  |
| Not in cluster | 982 | 87.8 | 294 | 67.6 | 168 | 78.9 | 338 | 73.8 | 827 | 73.7 | 100 | 24.9 | 97 | 23.0 | 165 | 21.6 | 181 | 28.7 |
| Neighbour of cluster | 12 | 2.7 | 19 | 4.4 | 3 | 1.4 | 22 | 4.8 | 28 | 2.5 | 24 | 6.0 | 30 | 7.1 | 45 | 5.9 | 35 | 5.5 |
| Left a cluster | 32 | 7.1 | 14 | 3.2 | 8 | 3.8 | 25 | 5.5 | 57 | 5.1 | 22 | 5.5 | 37 | 8.8 | 50 | 6.6 | 42 | 6.7 |
| Joined a cluster | 82 | 18.2 | 85 | 19.5 | 28 | 13.1 | 57 | 12.4 | 152 | 13.5 | 93 | 23.1 | 61 | 14.5 | 159 | 20.8 | 137 | 21.7 |
| Always been in cluster | 10 | 2.2 | 23 | 5.3 | 6 | 2.8 | 16 | 3.5 | 58 | 5.2 | 163 | 40.5 | 197 | 46.7 | 344 | 45.1 | 236 | 37.4 |
| **2007–2009** |  |  |  |  |  |  |  |  |  |  |  |  |  |  |  |  |  |  |
| Not in cluster | 978 | 87.5 | 289 | 66.4 | 165 | 77.5 | 328 | 71.6 | 801 | 71.4 | 94 | 23.4 | 93 | 22.0 | 147 | 19.3 | 163 | 25.8 |
| Neighbour of cluster | 7 | 1.6 | 15 | 3.4 | 3 | 1.4 | 13 | 2.8 | 21 | 1.9 | 16 | 4.0 | 18 | 4.3 | 31 | 4.1 | 28 | 4.4 |
| Left a cluster | 34 | 7.6 | 13 | 3.0 | 13 | 6.1 | 33 | 7.2 | 66 | 5.9 | 23 | 5.7 | 49 | 11.6 | 72 | 9.4 | 47 | 7.4 |
| Joined a cluster | 89 | 19.8 | 95 | 21.8 | 28 | 13.1 | 69 | 15.1 | 183 | 16.3 | 111 | 27.6 | 75 | 17.8 | 188 | 24.6 | 167 | 26.5 |
| Always been in cluster | 10 | 2.2 | 23 | 5.3 | 4 | 1.9 | 15 | 3.3 | 51 | 4.5 | 158 | 39.3 | 187 | 44.3 | 325 | 42.6 | 226 | 35.8 |
| **2009–2011** |  |  |  |  |  |  |  |  |  |  |  |  |  |  |  |  |  |  |
| Not in cluster | 973 | 87.0 | 286 | 65.7 | 161 | 75.6 | 320 | 69.9 | 787 | 70.1 | 90 | 22.4 | 84 | 19.9 | 141 | 18.5 | 158 | 25.0 |
| Neighbour of cluster | 4 | 0.9 | 8 | 1.8 | 4 | 1.9 | 12 | 2.6 | 14 | 1.2 | 10 | 2.5 | 16 | 3.8 | 19 | 2.5 | 18 | 2.9 |
| Left a cluster | 43 | 9.6 | 19 | 4.4 | 12 | 5.6 | 33 | 7.2 | 81 | 7.2 | 25 | 6.2 | 51 | 12.1 | 84 | 11.0 | 75 | 11.9 |
| Joined a cluster | 90 | 20.0 | 102 | 23.4 | 32 | 15.0 | 79 | 17.2 | 200 | 17.8 | 128 | 31.8 | 99 | 23.5 | 223 | 29.2 | 186 | 29.5 |
| Always been in cluster | 8 | 1.8 | 20 | 4.6 | 4 | 1.9 | 14 | 3.1 | 40 | 3.6 | 149 | 37.1 | 172 | 40.8 | 296 | 38.8 | 194 | 30.7 |
| **2011–2013** |  |  |  |  |  |  |  |  |  |  |  |  |  |  |  |  |  |  |
| Not in cluster | 970 | 86.8 | 280 | 64.4 | 158 | 74.2 | 318 | 69.4 | 780 | 69.5 | 83 | 20.6 | 82 | 19.4 | 127 | 16.6 | 153 | 24.2 |
| Neighbour of cluster | 3 | 0.7 | 7 | 1.6 | 4 | 1.9 | 6 | 1.3 | 9 | 0.8 | 7 | 1.7 | 13 | 3.1 | 12 | 1.6 | 15 | 2.4 |
| Left a cluster | 40 | 8.9 | 18 | 4.1 | 11 | 5.2 | 40 | 8.7 | 84 | 7.5 | 29 | 7.2 | 50 | 11.8 | 91 | 11.9 | 77 | 12.2 |
| Joined a cluster | 98 | 21.8 | 110 | 25.3 | 37 | 17.4 | 82 | 17.9 | 218 | 19.4 | 137 | 34.1 | 107 | 25.4 | 249 | 32.6 | 204 | 32.3 |
| Always been in cluster | 7 | 1.6 | 20 | 4.6 | 3 | 1.4 | 12 | 2.6 | 31 | 2.8 | 146 | 36.3 | 170 | 40.3 | 284 | 37.2 | 182 | 28.8 |
| **2013–2015** |  |  |  |  |  |  |  |  |  |  |  |  |  |  |  |  |  |  |
| Not in cluster | 962 | 86.0 | 279 | 64.1 | 157 | 73.7 | 311 | 67.9 | 770 | 68.6 | 80 | 19.9 | 80 | 19.0 | 118 | 15.5 | 145 | 23.0 |
| Neighbour of cluster | 3 | 0.7 | 6 | 1.4 | 3 | 1.4 | 6 | 1.3 | 6 | 0.5 | 7 | 1.7 | 12 | 2.8 | 9 | 1.2 | 14 | 2.2 |
| Left a cluster | 39 | 8.7 | 17 | 3.9 | 11 | 5.2 | 39 | 8.5 | 83 | 7.4 | 29 | 7.2 | 51 | 12.1 | 121 | 15.9 | 101 | 16.0 |
| Joined a cluster | 107 | 23.8 | 114 | 26.2 | 39 | 18.3 | 92 | 20.1 | 237 | 21.1 | 148 | 36.8 | 120 | 28.4 | 269 | 35.3 | 227 | 36.0 |
| Always been in cluster | 7 | 1.6 | 19 | 4.4 | 3 | 1.4 | 10 | 2.2 | 26 | 2.3 | 138 | 34.3 | 159 | 37.7 | 246 | 32.2 | 144 | 22.8 |
| **2015–2017** |  |  |  |  |  |  |  |  |  |  |  |  |  |  |  |  |  |  |
| Not in cluster | 952 | 85.2 | 262 | 60.2 | 151 | 70.9 | 304 | 66.4 | 751 | 66.9 | 68 | 16.9 | 74 | 17.5 | 106 | 13.9 | 133 | 21.1 |
| Neighbour of cluster | 2 | 0.4 | 4 | 0.9 | 3 | 1.4 | 4 | 0.9 | 7 | 0.6 | 5 | 1.2 | 10 | 2.4 | 7 | 0.9 | 10 | 1.6 |
| Left a cluster | 39 | 8.7 | 17 | 3.9 | 12 | 5.6 | 38 | 8.3 | 82 | 7.3 | 36 | 9.0 | 49 | 11.6 | 121 | 15.9 | 103 | 16.3 |
| Joined a cluster | 118 | 26.2 | 133 | 30.6 | 44 | 20.7 | 102 | 22.3 | 256 | 22.8 | 161 | 40.0 | 130 | 30.8 | 289 | 37.9 | 248 | 39.3 |
| Always been in cluster | 7 | 1.6 | 19 | 4.4 | 3 | 1.4 | 10 | 2.2 | 26 | 2.3 | 132 | 32.8 | 159 | 37.7 | 240 | 31.5 | 137 | 21.7 |
| **2017–2019** |  |  |  |  |  |  |  |  |  |  |  |  |  |  |  |  |  |  |
| Not in cluster | 941 | 84.2 | 250 | 57.5 | 148 | 69.5 | 294 | 64.2 | 725 | 64.6 | 57 | 14.2 | 53 | 12.6 | 87 | 11.4 | 116 | 18.4 |
| Neighbour of cluster | 2 | 0.4 | 1 | 0.2 | 3 | 1.4 | 4 | 0.9 | 3 | 0.3 | 1 | 0.2 | 2 | 0.5 | 2 | 0.3 | 4 | 0.6 |
| Left a cluster | 41 | 9.1 | 24 | 5.5 | 13 | 6.1 | 38 | 8.3 | 90 | 8.0 | 44 | 10.9 | 55 | 13.0 | 131 | 17.2 | 106 | 16.8 |
| Joined a cluster | 131 | 29.1 | 148 | 34.0 | 47 | 22.1 | 114 | 24.9 | 290 | 25.8 | 186 | 46.3 | 171 | 40.5 | 350 | 45.9 | 303 | 48.0 |
| Always been in cluster | 3 | 0.7 | 12 | 2.8 | 2 | 0.9 | 8 | 1.7 | 14 | 1.2 | 114 | 28.4 | 141 | 33.4 | 193 | 25.3 | 102 | 16.2 |
| **2019–2021** |  |  |  |  |  |  |  |  |  |  |  |  |  |  |  |  |  |  |
| Not in cluster | 933 | 83.5 | 247 | 56.8 | 147 | 69.0 | 292 | 63.8 | 714 | 63.6 | 55 | 13.7 | 51 | 12.1 | 82 | 10.7 | 114 | 18.1 |
| Neighbour of cluster | 2 | 0.4 | 2 | 0.5 | 2 | 0.9 | 3 | 0.7 | 3 | 0.3 | 2 | 0.5 | 1 | 0.2 | 0 | 0.0 | 3 | 0.5 |
| Left a cluster | 36 | 8.0 | 17 | 3.9 | 13 | 6.1 | 39 | 8.5 | 75 | 6.7 | 42 | 10.4 | 44 | 10.4 | 130 | 17.0 | 85 | 13.5 |
| Joined a cluster | 144 | 32.0 | 157 | 36.1 | 49 | 23.0 | 119 | 26.0 | 319 | 28.4 | 192 | 47.8 | 198 | 46.9 | 376 | 49.3 | 335 | 53.1 |
| Always been in cluster | 3 | 0.7 | 12 | 2.8 | 2 | 0.9 | 5 | 1.1 | 11 | 1.0 | 111 | 27.6 | 128 | 30.3 | 175 | 22.9 | 94 | 14.9 |
| **2021–2023** |  |  |  |  |  |  |  |  |  |  |  |  |  |  |  |  |  |  |
| Not in cluster | 919 | 82.2 | 246 | 56.6 | 143 | 67.1 | 286 | 62.4 | 705 | 62.8 | 55 | 13.7 | 51 | 12.1 | 80 | 10.5 | 114 | 18.1 |
| Neighbour of cluster | 2 | 0.4 | 0 | 0.0 | 2 | 0.9 | 3 | 0.7 | 4 | 0.4 | 1 | 0.2 | 1 | 0.2 | 0 | 0.0 | 3 | 0.5 |
| Left a cluster | 34 | 7.6 | 23 | 5.3 | 11 | 5.2 | 34 | 7.4 | 65 | 5.8 | 45 | 11.2 | 56 | 13.3 | 93 | 12.2 | 77 | 12.2 |
| Joined a cluster | 160 | 35.6 | 155 | 35.6 | 55 | 25.8 | 130 | 28.4 | 338 | 30.1 | 195 | 48.5 | 196 | 46.4 | 426 | 55.8 | 347 | 55.0 |
| Always been in cluster | 3 | 0.7 | 11 | 2.5 | 2 | 0.9 | 5 | 1.1 | 10 | 0.9 | 106 | 26.4 | 118 | 28.0 | 164 | 21.5 | 90 | 14.3 |

N: Number, %: Percentage.
