# Supplementary material for: Persistent and Emerging High‐Risk Clusters of Leprosy Detection in Brazil: A Nationwide Spatiotemporal Analysis, 2001–2023
Source: Trop Med Int Health. 2026 Feb 16;31(4):532–46. doi: 10.1111/tmi.70104 (PMC13050618; doi:10.1111/tmi.70104)
Supplement: Supplementary file 4 — Table S3: Classification of epidemiological and operational groups in the 2024–2030 Strategy in relation to clusters and their temporal evolution, Brazil, 2001–2023. [file TMI-31-532-s004.docx]

**Supplements – Table 3:** Classification of epidemiological and operational groups in the 2024–2030 Strategy in relation to clusters and their temporal evolution, Brazil, 2001–2023

| **Municipality classification** | **No cases** | | **Group 2.1** | | **Group 2.2** | | | | | | **Group 2.3** | | | | | |
| --- | --- | --- | --- | --- | --- | --- | --- | --- | --- | --- | --- | --- | --- | --- | --- | --- |
|  |  |  |  |  | **Subgroup 2.2.1** | | **Subgroup 2.2.2** | | **Subgroup 2.2.3** | | **Subgroup 2.3.1** | | **Subgroup 2.3.2** | | **Subgroup 2.3.3** | |
|  | **N** | **%** | **N** | **%** | **N** | **%** | **N** | **%** | **N** | **%** | **N** | **%** | **N** | **%** | **N** | **%** |
| **Number of municipalities** | **1,391** | **25.0** | **1,704** | **30.6** | **590** | **10.6** | **366** | **6.6** | **257** | **4.6** | **526** | **9.4** | **284** | **5.1** | **452** | **8.1** |
| **Ranking** |  |  |  |  |  |  |  |  |  |  |  |  |  |  |  |  |
| **2001–2003** |  |  |  |  |  |  |  |  |  |  |  |  |  |  |  |  |
| Not clustered and not neighbour | 1,274 | 91.6 | 1,292 | 75.8 | 330 | 55.9 | 229 | 62.6 | 158 | 61.5 | 189 | 35.9 | 116 | 40.8 | 143 | 31.6 |
| Not significant cluster neighbour | 8 | 1.8 | 20 | 1.2 | 7 | 1.2 | 3 | 0.8 | 3 | 1.2 | 3 | 0.6 | 2 | 0.7 | 7 | 1.5 |
| Not significant cluster | 0 | 0.0 | 8 | 0.5 | 1 | 0.2 | 0 | 0.0 | 2 | 0.8 | 3 | 0.6 | 1 | 0.4 | 5 | 1.1 |
| Significant cluster neighbour | 74 | 16.4 | 180 | 10.6 | 78 | 13.2 | 46 | 12.6 | 19 | 7.4 | 76 | 14.4 | 33 | 11.6 | 47 | 10.4 |
| Significant cluster | 35 | 7.8 | 204 | 12.0 | 174 | 29.5 | 88 | 24.0 | 75 | 29.2 | 255 | 48.5 | 132 | 46.5 | 250 | 55.3 |
| **2003–2005** |  |  |  |  |  |  |  |  |  |  |  |  |  |  |  |  |
| Not clustered and not neighbour | 1,255 | 90.2 | 1,233 | 72.4 | 282 | 47.8 | 203 | 55.5 | 129 | 50.2 | 151 | 28.7 | 96 | 33.8 | 116 | 25.7 |
| Not significant cluster neighbour | 11 | 2.4 | 37 | 2.2 | 11 | 1.9 | 6 | 1.6 | 4 | 1.6 | 14 | 2.7 | 2 | 0.7 | 10 | 2.2 |
| Not significant cluster | 2 | 0.4 | 7 | 0.4 | 9 | 1.5 | 2 | 0.5 | 5 | 1.9 | 5 | 1.0 | 5 | 1.8 | 1 | 0.2 |
| Significant cluster neighbour | 64 | 14.2 | 153 | 9.0 | 82 | 13.9 | 52 | 14.2 | 30 | 11.7 | 59 | 11.2 | 34 | 12.0 | 58 | 12.8 |
| Significant cluster | 59 | 13.1 | 274 | 16.1 | 206 | 34.9 | 103 | 28.1 | 89 | 34.6 | 297 | 56.5 | 147 | 51.8 | 267 | 59.1 |
| **2005–2007** |  |  |  |  |  |  |  |  |  |  |  |  |  |  |  |  |
| Not clustered and not neighbour | 1,262 | 90.7 | 1,256 | 73.7 | 292 | 49.5 | 217 | 59.3 | 147 | 57.2 | 159 | 30.2 | 104 | 36.6 | 130 | 28.8 |
| Not significant cluster neighbour | 21 | 4.7 | 33 | 1.9 | 11 | 1.9 | 4 | 1.1 | 4 | 1.6 | 3 | 0.6 | 1 | 0.4 | 3 | 0.7 |
| Not significant cluster | 1 | 0.2 | 6 | 0.4 | 6 | 1.0 | 1 | 0.3 | 2 | 0.8 | 3 | 0.6 | 2 | 0.7 | 3 | 0.7 |
| Significant cluster neighbour | 55 | 12.2 | 168 | 9.9 | 81 | 13.7 | 48 | 13.1 | 30 | 11.7 | 71 | 13.5 | 33 | 11.6 | 65 | 14.4 |
| Significant cluster | 52 | 11.6 | 241 | 14.1 | 200 | 33.9 | 96 | 26.2 | 74 | 28.8 | 290 | 55.1 | 144 | 50.7 | 251 | 55.5 |
| **2007–2009** |  |  |  |  |  |  |  |  |  |  |  |  |  |  |  |  |
| Not clustered and not neighbour | 1,271 | 91.4 | 1,252 | 73.5 | 293 | 49.7 | 221 | 60.4 | 144 | 56.0 | 162 | 30.8 | 106 | 37.3 | 140 | 31.0 |
| Not significant cluster neighbour | 20 | 4.4 | 41 | 2.4 | 14 | 2.4 | 14 | 3.8 | 9 | 3.5 | 5 | 1.0 | 3 | 1.1 | 5 | 1.1 |
| Not significant cluster | 3 | 0.7 | 15 | 0.9 | 12 | 2.0 | 2 | 0.5 | 4 | 1.6 | 2 | 0.4 | 1 | 0.4 | 3 | 0.7 |
| Significant cluster neighbour | 35 | 7.8 | 141 | 8.3 | 63 | 10.7 | 36 | 9.8 | 24 | 9.3 | 80 | 15.2 | 33 | 11.6 | 57 | 12.6 |
| Significant cluster | 62 | 13.8 | 255 | 15.0 | 208 | 35.3 | 93 | 25.4 | 76 | 29.6 | 277 | 52.7 | 141 | 49.6 | 247 | 54.6 |
| **2009–2011** |  |  |  |  |  |  |  |  |  |  |  |  |  |  |  |  |
| Not clustered and not neighbour | 1,287 | 92.5 | 1,299 | 76.2 | 305 | 51.7 | 220 | 60.1 | 145 | 56.4 | 166 | 31.6 | 101 | 35.6 | 141 | 31.2 |
| Not significant cluster neighbour | 14 | 3.1 | 31 | 1.8 | 13 | 2.2 | 6 | 1.6 | 4 | 1.6 | 7 | 1.3 | 6 | 2.1 | 7 | 1.5 |
| Not significant cluster | 0 | 0.0 | 3 | 0.2 | 5 | 0.8 | 2 | 0.5 | 2 | 0.8 | 3 | 0.6 | 1 | 0.4 | 2 | 0.4 |
| Significant cluster neighbour | 38 | 8.4 | 127 | 7.5 | 72 | 12.2 | 43 | 11.7 | 33 | 12.8 | 79 | 15.0 | 34 | 12.0 | 61 | 13.5 |
| Significant cluster | 52 | 11.6 | 244 | 14.3 | 195 | 33.1 | 95 | 26.0 | 73 | 28.4 | 271 | 51.5 | 142 | 50.0 | 241 | 53.3 |
| **2011–2013** |  |  |  |  |  |  |  |  |  |  |  |  |  |  |  |  |
| Not clustered and not neighbour | 1,299 | 93.4 | 1,334 | 78.3 | 311 | 52.7 | 215 | 58.7 | 156 | 60.7 | 170 | 32.3 | 113 | 39.8 | 151 | 33.4 |
| Not significant cluster neighbour | 7 | 1.6 | 24 | 1.4 | 14 | 2.4 | 5 | 1.4 | 2 | 0.8 | 8 | 1.5 | 3 | 1.1 | 5 | 1.1 |
| Not significant cluster | 1 | 0.2 | 6 | 0.4 | 10 | 1.7 | 1 | 0.3 | 2 | 0.8 | 4 | 0.8 | 4 | 1.4 | 2 | 0.4 |
| Significant cluster neighbour | 42 | 9.3 | 123 | 7.2 | 68 | 11.5 | 39 | 10.7 | 29 | 11.3 | 63 | 12.0 | 31 | 10.9 | 55 | 12.2 |
| Significant cluster | 42 | 9.3 | 217 | 12.7 | 187 | 31.7 | 106 | 29.0 | 68 | 26.5 | 281 | 53.4 | 133 | 46.8 | 239 | 52.9 |
| **2013–2015** |  |  |  |  |  |  |  |  |  |  |  |  |  |  |  |  |
| Not clustered and not neighbour | 1,290 | 92.7 | 1,325 | 77.8 | 321 | 54.4 | 226 | 61.7 | 151 | 58.8 | 180 | 34.2 | 105 | 37.0 | 162 | 35.8 |
| Not significant cluster neighbour | 8 | 1.8 | 21 | 1.2 | 8 | 1.4 | 4 | 1.1 | 2 | 0.8 | 3 | 0.6 | 4 | 1.4 | 0 | 0.0 |
| Not significant cluster | 3 | 0.7 | 4 | 0.2 | 4 | 0.7 | 1 | 0.3 | 2 | 0.8 | 2 | 0.4 | 1 | 0.4 | 0 | 0.0 |
| Significant cluster neighbour | 40 | 8.9 | 120 | 7.0 | 77 | 13.1 | 43 | 11.7 | 29 | 11.3 | 77 | 14.6 | 34 | 12.0 | 55 | 12.2 |
| Significant cluster | 50 | 11.1 | 234 | 13.7 | 180 | 30.5 | 92 | 25.1 | 73 | 28.4 | 264 | 50.2 | 140 | 49.3 | 235 | 52.0 |
| **2015–2017** |  |  |  |  |  |  |  |  |  |  |  |  |  |  |  |  |
| Not clustered and not neighbour | 1,292 | 92.9 | 1,298 | 76.2 | 321 | 54.4 | 215 | 58.7 | 141 | 54.9 | 157 | 29.8 | 97 | 34.2 | 155 | 34.3 |
| Not significant cluster neighbour | 3 | 0.7 | 16 | 0.9 | 7 | 1.2 | 0 | 0.0 | 5 | 1.9 | 1 | 0.2 | 1 | 0.4 | 0 | 0.0 |
| Not significant cluster | 0 | 0.0 | 4 | 0.2 | 2 | 0.3 | 0 | 0.0 | 0 | 0.0 | 3 | 0.6 | 2 | 0.7 | 0 | 0.0 |
| Significant cluster neighbour | 42 | 9.3 | 112 | 6.6 | 60 | 10.2 | 40 | 10.9 | 35 | 13.6 | 75 | 14.3 | 31 | 10.9 | 55 | 12.2 |
| Significant cluster | 54 | 12.0 | 274 | 16.1 | 200 | 33.9 | 111 | 30.3 | 76 | 29.6 | 290 | 55.1 | 153 | 53.9 | 242 | 53.5 |
| **2017–2019** |  |  |  |  |  |  |  |  |  |  |  |  |  |  |  |  |
| Not clustered and not neighbour | 1,304 | 93.7 | 1,305 | 76.6 | 326 | 55.3 | 223 | 60.9 | 158 | 61.5 | 180 | 34.2 | 115 | 40.5 | 145 | 32.1 |
| Not significant cluster neighbour | 4 | 0.9 | 20 | 1.2 | 6 | 1.0 | 2 | 0.5 | 2 | 0.8 | 5 | 1.0 | 2 | 0.7 | 7 | 1.5 |
| Not significant cluster | 0 | 0.0 | 0 | 0.0 | 1 | 0.2 | 0 | 0.0 | 1 | 0.4 | 2 | 0.4 | 1 | 0.4 | 4 | 0.9 |
| Significant cluster neighbour | 34 | 7.6 | 121 | 7.1 | 76 | 12.9 | 35 | 9.6 | 23 | 8.9 | 58 | 11.0 | 38 | 13.4 | 50 | 11.1 |
| Significant cluster | 49 | 10.9 | 258 | 15.1 | 181 | 30.7 | 106 | 29.0 | 73 | 28.4 | 281 | 53.4 | 128 | 45.1 | 246 | 54.4 |
| **2019–2021** |  |  |  |  |  |  |  |  |  |  |  |  |  |  |  |  |
| Not clustered and not neighbour | 1,324 | 95.2 | 1,377 | 80.8 | 351 | 59.5 | 231 | 63.1 | 176 | 68.5 | 192 | 36.5 | 123 | 43.3 | 162 | 35.8 |
| Not significant cluster neighbour | 1 | 0.2 | 12 | 0.7 | 2 | 0.3 | 0 | 0.0 | 0 | 0.0 | 8 | 1.5 | 2 | 0.7 | 2 | 0.4 |
| Not significant cluster | 7 | 1.6 | 12 | 0.7 | 4 | 0.7 | 1 | 0.3 | 0 | 0.0 | 5 | 1.0 | 0 | 0.0 | 4 | 0.9 |
| Significant cluster neighbour | 31 | 6.9 | 92 | 5.4 | 75 | 12.7 | 35 | 9.6 | 18 | 7.0 | 63 | 12.0 | 39 | 13.7 | 49 | 10.8 |
| Significant cluster | 28 | 6.2 | 211 | 12.4 | 158 | 26.8 | 99 | 27.0 | 63 | 24.5 | 258 | 49.0 | 120 | 42.3 | 235 | 52.0 |
| **2021–2023** |  |  |  |  |  |  |  |  |  |  |  |  |  |  |  |  |
| Not clustered and not neighbour | 1,331 | 95.7 | 1,437 | 84.3 | 392 | 66.4 | 261 | 71.3 | 188 | 73.2 | 245 | 46.6 | 149 | 52.5 | 197 | 43.6 |
| Not significant cluster neighbour | 9 | 2.0 | 28 | 1.6 | 11 | 1.9 | 8 | 2.2 | 2 | 0.8 | 4 | 0.8 | 6 | 2.1 | 6 | 1.3 |
| Not significant cluster | 1 | 0.2 | 8 | 0.5 | 4 | 0.7 | 4 | 1.1 | 2 | 0.8 | 8 | 1.5 | 2 | 0.7 | 6 | 1.3 |
| Significant cluster neighbour | 26 | 5.8 | 78 | 4.6 | 49 | 8.3 | 26 | 7.1 | 17 | 6.6 | 66 | 12.5 | 20 | 7.0 | 40 | 8.8 |
| Significant cluster | 24 | 5.3 | 153 | 9.0 | 134 | 22.7 | 67 | 18.3 | 48 | 18.7 | 203 | 38.6 | 107 | 37.7 | 203 | 44.9 |
| **Ranking – comparison** |  |  |  |  |  |  |  |  |  |  |  |  |  |  |  |  |
| **2001–2003** |  |  |  |  |  |  |  |  |  |  |  |  |  |  |  |  |
| Not in cluster | - |  | - |  | - |  | - |  | - |  | - | - | - | - | - | - |
| Neighbour of cluster | - |  | - |  | - |  | - |  | - |  | - | - | - | - | - | - |
| Left a cluster | - |  | - |  | - |  | - |  | - |  | - | - | - | - | - | - |
| Joined a cluster | - |  | - |  | - |  | - |  | - |  | - | - | - | - | - | - |
| Always been in cluster | - |  | - |  | - |  | - |  | - |  | - | - | - | - | - | - |
| **2003–2005** |  |  |  |  |  |  |  |  |  |  |  |  |  |  |  |  |
| Not in cluster | 1,222 | 87.9 | 1,156 | 67.8 | 265 | 44.9 | 193 | 52.7 | 124 | 48.2 | 131 | 24.9 | 88 | 31.0 | 105 | 23.2 |
| Neighbour of cluster | 29 | 6.4 | 77 | 4.5 | 42 | 7.1 | 29 | 7.9 | 10 | 3.9 | 38 | 7.2 | 20 | 7.0 | 30 | 6.6 |
| Left a cluster | 44 | 9.8 | 121 | 7.1 | 38 | 6.4 | 22 | 6.0 | 13 | 5.1 | 34 | 6.5 | 17 | 6.0 | 34 | 7.5 |
| Joined a cluster | 69 | 15.3 | 178 | 10.4 | 88 | 14.9 | 44 | 12.0 | 42 | 16.3 | 82 | 15.6 | 37 | 13.0 | 55 | 12.2 |
| Always been in cluster | 27 | 6.0 | 172 | 10.1 | 157 | 26.6 | 78 | 21.3 | 68 | 26.5 | 241 | 45.8 | 122 | 43.0 | 228 | 50.4 |
| **2005–2007** |  |  |  |  |  |  |  |  |  |  |  |  |  |  |  |  |
| Not in cluster | 1,203 | 86.5 | 1,116 | 65.5 | 240 | 40.7 | 177 | 48.4 | 121 | 47.1 | 118 | 22.4 | 80 | 28.2 | 100 | 22.1 |
| Neighbour of cluster | 21 | 4.7 | 59 | 3.5 | 35 | 5.9 | 25 | 6.8 | 7 | 2.7 | 32 | 6.1 | 14 | 4.9 | 25 | 5.5 |
| Left a cluster | 39 | 8.7 | 96 | 5.6 | 29 | 4.9 | 23 | 6.3 | 22 | 8.6 | 24 | 4.6 | 19 | 6.7 | 35 | 7.7 |
| Joined a cluster | 103 | 22.9 | 270 | 15.8 | 130 | 22.0 | 66 | 18.0 | 47 | 18.3 | 114 | 21.7 | 54 | 19.0 | 71 | 15.7 |
| Always been in cluster | 25 | 5.6 | 163 | 9.6 | 156 | 26.4 | 75 | 20.5 | 60 | 23.3 | 238 | 45.2 | 117 | 41.2 | 221 | 48.9 |
| **2007–2009** |  |  |  |  |  |  |  |  |  |  |  |  |  |  |  |  |
| Not in cluster | 1,185 | 85.2 | 1,082 | 63.5 | 224 | 38.0 | 171 | 46.7 | 113 | 44.0 | 110 | 20.9 | 79 | 27.8 | 97 | 21.5 |
| Neighbour of cluster | 10 | 2.2 | 46 | 2.7 | 23 | 3.9 | 19 | 5.2 | 3 | 1.2 | 24 | 4.6 | 10 | 3.5 | 17 | 3.8 |
| Left a cluster | 46 | 10.2 | 97 | 5.7 | 38 | 6.4 | 30 | 8.2 | 24 | 9.3 | 40 | 7.6 | 24 | 8.5 | 51 | 11.3 |
| Joined a cluster | 126 | 28.0 | 323 | 19.0 | 155 | 26.3 | 74 | 20.2 | 58 | 22.6 | 131 | 24.9 | 60 | 21.1 | 79 | 17.5 |
| Always been in cluster | 24 | 5.3 | 156 | 9.2 | 150 | 25.4 | 72 | 19.7 | 59 | 23.0 | 221 | 42.0 | 111 | 39.1 | 208 | 46.0 |
| **2009–2011** |  |  |  |  |  |  |  |  |  |  |  |  |  |  |  |  |
| Not in cluster | 1,179 | 84.8 | 1,062 | 62.3 | 218 | 36.9 | 165 | 45.1 | 109 | 42.4 | 105 | 20.0 | 73 | 25.7 | 92 | 20.4 |
| Neighbour of cluster | 7 | 1.6 | 30 | 1.8 | 14 | 2.4 | 13 | 3.6 | 3 | 1.2 | 15 | 2.9 | 8 | 2.8 | 15 | 3.3 |
| Left a cluster | 53 | 11.8 | 123 | 7.2 | 54 | 9.2 | 35 | 9.6 | 26 | 10.1 | 55 | 10.5 | 25 | 8.8 | 52 | 11.5 |
| Joined a cluster | 130 | 28.9 | 353 | 20.7 | 177 | 30.0 | 93 | 25.4 | 67 | 26.1 | 150 | 28.5 | 76 | 26.8 | 94 | 20.8 |
| Always been in cluster | 22 | 4.9 | 136 | 8.0 | 127 | 21.5 | 60 | 16.4 | 52 | 20.2 | 201 | 38.2 | 102 | 35.9 | 199 | 44.0 |
| **2011–2013** |  |  |  |  |  |  |  |  |  |  |  |  |  |  |  |  |
| Not in cluster | 1,178 | 84.7 | 1,049 | 61.6 | 211 | 35.8 | 152 | 41.5 | 106 | 41.2 | 102 | 19.4 | 70 | 24.6 | 86 | 19.0 |
| Neighbour of cluster | 6 | 1.3 | 18 | 1.1 | 8 | 1.4 | 10 | 2.7 | 3 | 1.2 | 13 | 2.5 | 8 | 2.8 | 10 | 2.2 |
| Left a cluster | 51 | 11.3 | 129 | 7.6 | 62 | 10.5 | 36 | 9.8 | 24 | 9.3 | 51 | 9.7 | 23 | 8.1 | 64 | 14.2 |
| Joined a cluster | 139 | 30.9 | 379 | 22.2 | 191 | 32.4 | 110 | 30.1 | 74 | 28.8 | 167 | 31.7 | 83 | 29.2 | 100 | 22.1 |
| Always been in cluster | 17 | 3.8 | 129 | 7.6 | 118 | 20.0 | 58 | 15.8 | 50 | 19.5 | 193 | 36.7 | 100 | 35.2 | 192 | 42.5 |
| **2013–2015** |  |  |  |  |  |  |  |  |  |  |  |  |  |  |  |  |
| Not in cluster | 1,172 | 84.3 | 1,028 | 60.3 | 204 | 34.6 | 148 | 40.4 | 103 | 40.1 | 100 | 19.0 | 66 | 23.2 | 84 | 18.6 |
| Neighbour of cluster | 6 | 1.3 | 17 | 1.0 | 8 | 1.4 | 6 | 1.6 | 2 | 0.8 | 13 | 2.5 | 7 | 2.5 | 7 | 1.5 |
| Left a cluster | 53 | 11.8 | 143 | 8.4 | 77 | 13.1 | 40 | 10.9 | 29 | 11.3 | 55 | 10.5 | 27 | 9.5 | 67 | 14.8 |
| Joined a cluster | 144 | 32.0 | 407 | 23.9 | 205 | 34.7 | 124 | 33.9 | 79 | 30.7 | 184 | 35.0 | 95 | 33.5 | 116 | 25.7 |
| Always been in cluster | 16 | 3.6 | 109 | 6.4 | 96 | 16.3 | 48 | 13.1 | 44 | 17.1 | 174 | 33.1 | 89 | 31.3 | 178 | 39.4 |
| **2015–2017** |  |  |  |  |  |  |  |  |  |  |  |  |  |  |  |  |
| Not in cluster | 1,163 | 83.6 | 995 | 58.4 | 187 | 31.7 | 139 | 38.0 | 94 | 36.6 | 86 | 16.3 | 61 | 21.5 | 79 | 17.5 |
| Neighbour of cluster | 4 | 0.9 | 13 | 0.8 | 4 | 0.7 | 3 | 0.8 | 2 | 0.8 | 13 | 2.5 | 6 | 2.1 | 7 | 1.5 |
| Left a cluster | 60 | 13.3 | 143 | 8.4 | 72 | 12.2 | 43 | 11.7 | 27 | 10.5 | 52 | 9.9 | 32 | 11.3 | 68 | 15.0 |
| Joined a cluster | 149 | 33.1 | 447 | 26.2 | 233 | 39.5 | 133 | 36.3 | 92 | 35.8 | 204 | 38.8 | 100 | 35.2 | 124 | 27.4 |
| Always been in cluster | 15 | 3.3 | 106 | 6.2 | 94 | 15.9 | 48 | 13.1 | 42 | 16.3 | 171 | 32.5 | 85 | 29.9 | 174 | 38.5 |
| **2017–2019** |  |  |  |  |  |  |  |  |  |  |  |  |  |  |  |  |
| Not in cluster | 1,148 | 82.5 | 953 | 55.9 | 168 | 28.5 | 127 | 34.7 | 87 | 33.9 | 69 | 13.1 | 53 | 18.7 | 69 | 15.3 |
| Neighbour of cluster | 3 | 0.7 | 7 | 0.4 | 2 | 0.3 | 1 | 0.3 | 0 | 0.0 | 1 | 0.2 | 4 | 1.4 | 4 | 0.9 |
| Left a cluster | 65 | 14.4 | 141 | 8.3 | 78 | 13.2 | 39 | 10.7 | 27 | 10.5 | 77 | 14.6 | 44 | 15.5 | 71 | 15.7 |
| Joined a cluster | 168 | 37.3 | 522 | 30.6 | 272 | 46.1 | 157 | 42.9 | 108 | 42.0 | 239 | 45.4 | 116 | 40.8 | 159 | 35.2 |
| Always been in cluster | 7 | 1.6 | 81 | 4.8 | 70 | 11.9 | 42 | 11.5 | 35 | 13.6 | 140 | 26.6 | 67 | 23.6 | 149 | 33.0 |
| **2019–2021** |  |  |  |  |  |  |  |  |  |  |  |  |  |  |  |  |
| Not in cluster | 1,140 | 82.0 | 934 | 54.8 | 164 | 27.8 | 126 | 34.4 | 85 | 33.1 | 69 | 13.1 | 53 | 18.7 | 67 | 14.8 |
| Neighbour of cluster | 2 | 0.4 | 7 | 0.4 | 2 | 0.3 | 0 | 0.0 | 0 | 0.0 | 1 | 0.2 | 3 | 1.1 | 3 | 0.7 |
| Left a cluster | 54 | 12.0 | 115 | 6.7 | 68 | 11.5 | 42 | 11.5 | 25 | 9.7 | 71 | 13.5 | 38 | 13.4 | 69 | 15.3 |
| Joined a cluster | 188 | 41.8 | 571 | 33.5 | 293 | 49.7 | 162 | 44.3 | 114 | 44.4 | 255 | 48.5 | 130 | 45.8 | 177 | 39.2 |
| Always been in cluster | 7 | 1.6 | 77 | 4.5 | 63 | 10.7 | 36 | 9.8 | 33 | 12.8 | 130 | 24.7 | 60 | 21.1 | 136 | 30.1 |
| **2021–2023** |  |  |  |  |  |  |  |  |  |  |  |  |  |  |  |  |
| Not in cluster | 1,125 | 80.9 | 924 | 54.2 | 160 | 27.1 | 126 | 34.4 | 80 | 31.1 | 69 | 13.1 | 51 | 18.0 | 67 | 14.8 |
| Neighbour of cluster | 2 | 0.4 | 6 | 0.4 | 2 | 0.3 | 0 | 0.0 | 0 | 0.0 | 1 | 0.2 | 2 | 0.7 | 3 | 0.7 |
| Left a cluster | 55 | 12.2 | 109 | 6.4 | 61 | 10.3 | 29 | 7.9 | 25 | 9.7 | 65 | 12.4 | 28 | 9.9 | 66 | 14.6 |
| Joined a cluster | 202 | 44.9 | 597 | 35.0 | 305 | 51.7 | 177 | 48.4 | 122 | 47.5 | 266 | 50.6 | 147 | 51.8 | 188 | 41.6 |
| Always been in cluster | 7 | 1.6 | 68 | 4.0 | 62 | 10.5 | 34 | 9.3 | 30 | 11.7 | 125 | 23.8 | 56 | 19.7 | 128 | 28.3 |

N: Number, %: Percentage.
